# Methodological Framework for the Design and Implementation of a US Latine-Hispanic Digital Brain Health Program: User-Centered Design Approach

**DOI:** 10.2196/73445

**Published:** 2026-05-14

**Authors:** Aisha Mohammed, Stephanie Ovalle-Eliseo, Jasmine Mohammed, Gabriela Islas Huerta, Lorena Monserratt, Diana Andrade, Jasmine Garcia, Raquel Kaufman, Marianne Gutierrez, Mirella Díaz-Santos

**Affiliations:** 1 Mary S. Easton Center for Alzheimer’s Research and Care, Equity for Latinx-Hispanic Healthy Aging (ELHA) Lab, University of California, Los Angeles, 710 Westwood Plaza, Room C-224, Los Angeles, CA, 90095-1769, United States, 1 310 794 0292; 2 Mary S. Easton Center for Alzheimer’s Research and Care, The Katherine & Benjamin Kagan Alzheimer's Disease Treatment Program, University of California, Los Angeles, Los Angeles, CA, United States

**Keywords:** brain health, digital education, social media, human-centered design, Spanish-English bilingual

## Abstract

**Background:**

US Latine and Hispanic communities face a 1.5 times greater risk of developing Alzheimer disease and related dementia (ADRD) with limited access to culturally and linguistically congruent primary prevention education. The COVID-19 pandemic exacerbated the digital divide, highlighting a need to focus on alternative digital methods for delivering brain health and ADRD primary prevention education. Social media emerged as a promising tool.

**Objective:**

The objective of this paper is two-fold. We first describe the development and pilot study of our social media–based Latine-Hispanic Digital Brain Health Program guided by evidence-based frameworks in ADRD. We then present the quantitative and qualitative results from the first 14 months of the program (October 2023-December 2024).

**Methods:**

We used human-centered design to develop the Digital Alzheimer Health Education Model, which was implemented via 3 social media platforms—Facebook, Instagram, and X (formerly known as Twitter). Our bilingual and bicultural team implemented the model by creating and disseminating tailored educational content in English and Spanish for the resulting Latine-Hispanic Digital Brain Health Program, emphasizing consistency and rapport, storytelling, cultural relevance, linguistic inclusivity, and visual representation. A mixed methods analysis (descriptive statistics and sentiment analysis) was conducted using social media data analytics and users’ comments to guide program evaluation and refinement.

**Results:**

From October 2023 to December 2024, we retained 857 followers across our social media platforms (Instagram: n=534; Facebook: n=124; and X: n=199). Growth in follows, consistent reach and engagement, and positive sentiment were observed on Facebook and Instagram. X was not included in the analysis due to data access limitations.

**Conclusions:**

The development and pilot study of the Latine-Hispanic Digital Brain Health Program have demonstrated potential in leveraging social media to disseminate brain health and ADRD prevention education to the US Latine and Hispanic communities in English and Spanish. Our preliminary findings demonstrate that culturally and linguistically congruent social media–based approaches hold potential to improve engagement with brain health and ADRD primary prevention education among US Latine and Hispanic populations.

## Introduction

### Alzheimer Disease in Latine and Hispanic Communities

Alzheimer disease and related dementia (ADRD) are some of the most prevalent and costly neurodegenerative conditions with no known cure [1]. In 2024, approximately 6.9 million American people aged 65+ years lived with ADRD, with the total cost of care, treatment, and prevention in the United States projected to surpass US $1 trillion by 2050 [1,2]. US Latine and Hispanic communities experience a 1.5 times increased risk of developing ADRD compared to non-Hispanic White individuals and face disparities in ADRD detection, diagnosis, treatment, and care management [1,3-9]. Current ADRD treatments focus on symptom management (ie, memory, language, and thinking), while emerging disease-modifying therapies provide only modest benefits without halting disease progression [10,11]. Given these constraints, prevention is critical [11-17]. The 2024 Lancet Commission identified 14 modifiable risk factors of ADRD (eg, hypertension, diabetes, and social isolation) that contribute to cognitive decline and dementia risk, many of which disproportionately affect US Latine and Hispanic communities [3,7,15,18,19]. A need to refocus efforts on primary prevention to reduce ADRD risk and alleviate personal, structural, and economic burdens while improving brain health outcomes is urgent, particularly for US Latine and Hispanic communities [12,13,15].

### Health Engagement in Latine and Hispanic Communities

Before the COVID-19 pandemic, in-person ADRD health promotion campaigns for US Latine and Hispanic communities were the most common and effective educational strategy for encouraging primary prevention [20-23]. These initiatives implemented evidence-based community engagement strategies, including community health worker involvement, and established community-based health promotion programs [20-22,24-26]. Moukarzel et al [21,22] demonstrated that in-person engagement through Spanish programs like “Hispanos y el ALTo a la Demencia [Healthy Actions and Lifestyles to Avoid Dementia],” with culturally tailored education and resources, significantly contributed to mitigating ADRD risk among US Latine and Hispanic communities. Face-to-face interactions in the program were particularly effective in building trust and delivering personalized guidance, helping participants overcome barriers and adopt primary prevention strategies [21,22]. These in-person strategies, ranging from community health worker involvement to tailored community-based programs, have increased engagement, trust, and participation in ADRD-related education and clinical research among US Latine and Hispanic communities [20-22,24-26].

The COVID-19 pandemic, however, forced changes in traditional health engagement practices with US Latine and Hispanic populations. Shifts to online education through websites and virtual Zoom (Zoom Video Communications) classes exposed and exacerbated the digital divide in ADRD health promotion efforts with these communities [27-33]. The digital divide refers to the gap between those with and without adequate access to information via technology [27-33]. This disparity has contributed to US Latine and Hispanic communities having less access to brain health and ADRD prevention information, and resources made available by nonprofits, government-based organizations, and academic institutions. For example, Gutiérrez et al [30] found that only 3.83% (8/209) of Latine and Hispanic individuals in their Southern California study participated in ADRD online education during the COVID-19 pandemic. They attributed the low participation to structural barriers such as limited high-speed internet access, digital literacy challenges, and financial constraints, all of which disproportionately affect US Latine and Hispanic populations [30]. Others found that additional factors such as age, socioeconomic status, and English-language proficiency further exacerbated disparities in digital health engagement [27-33]. Ovalle-Eliseo et al [27] also noted limitations to web navigation and accessibility for Latine and Hispanic people, fueled by the limited availability of Spanish-language content, inaccurate translations, and a lack of web accessibility tools. These findings highlight the need to explore other digital platforms that can enhance engagement for US Latine and Hispanic communities of all intersectionalities.

### Social Media as an Emerging Health Communication Tool

Social media, like Facebook, Instagram, and X (formerly known as Twitter), surfaced as feasible and acceptable platforms for delivering education during the COVID-19 pandemic, offering an opportunity to engage US Latine and Hispanic communities in digital health education. Approximately 85% of US Latine and Hispanic individuals use social media broadly [34,35]. In 2024, Pew Research found that 69% of US Latine and Hispanic individuals used Facebook, 59% used Instagram, and 22% used X [36]. This is attributed to the accessibility and reach of social media, making it particularly impactful for culturally and linguistically congruent health communication strategies in the field of public health [37-42] and, more recently, in the field of ADRD [43-47].

With regard to ADRD, one study demonstrated how caregivers used social media platforms to share experiences, provide support, and exchange caregiving tips, highlighting its potential for digital community-building [43]. Similarly, another study conducted by Teano et al [44,45] examined social media’s efficacy as an ADRD outreach and research recruitment strategy, highlighting its ability to reach diverse audiences. Despite these positive insights on social media’s feasibility and acceptance in engaging communities in ADRD research, neither study provided information on how the platforms could be leveraged for community-centered education. While Isaacson et al [46] did evaluate the use of social media in ADRD education, the authors focused on its use for recruitment, driving traffic to a website-based education program. Altogether, these studies provide an opportunity to leverage their insights on social media engagement strategies to explore these platforms’ potential in directly disseminating culturally and linguistically congruent brain health and ADRD information for primary prevention to US Latine and Hispanic communities.

### Evidence-Based Brain Health and ADRD Education for Latine and Hispanic Communities

To date, no social media study has implemented evidence-based frameworks in ADRD research, such as cognitive reserve theory [13,48-57], social or structural determinants of health (SDOH) [7,58-62] in ADRD, and human aging neuroscience to educate US Latine and Hispanic people on brain health and ADRD primary prevention in English and Spanish. By leveraging social media, digital health promotion campaigns grounded in evidence-based frameworks can potentially shape positive perceptions about brain health, ignite behavior change, and scale ADRD primary prevention efforts. Additionally, incorporating culturally relevant messaging and delivery that resonate with US Latine and Hispanic communities can increase the acceptance and relevance of these digital brain health programs [34,63-66].

The objective of this paper is 2-fold. We first describe the development and pilot study of our Latine-Hispanic Digital Brain Health Program across 3 social media platforms—Facebook, Instagram, and X—using evidence-based frameworks for ADRD. We then present the quantitative and qualitative results from the first 14 months. By outlining our process, strategy, and outcomes, we aim to offer a replicable model that other brain health and ADRD health educators can use to disseminate information that is culturally and linguistically tailored for Latine and Hispanic communities in the United States.

## Methods

### Conceptual Framework

#### Overview

**Figure 1.**
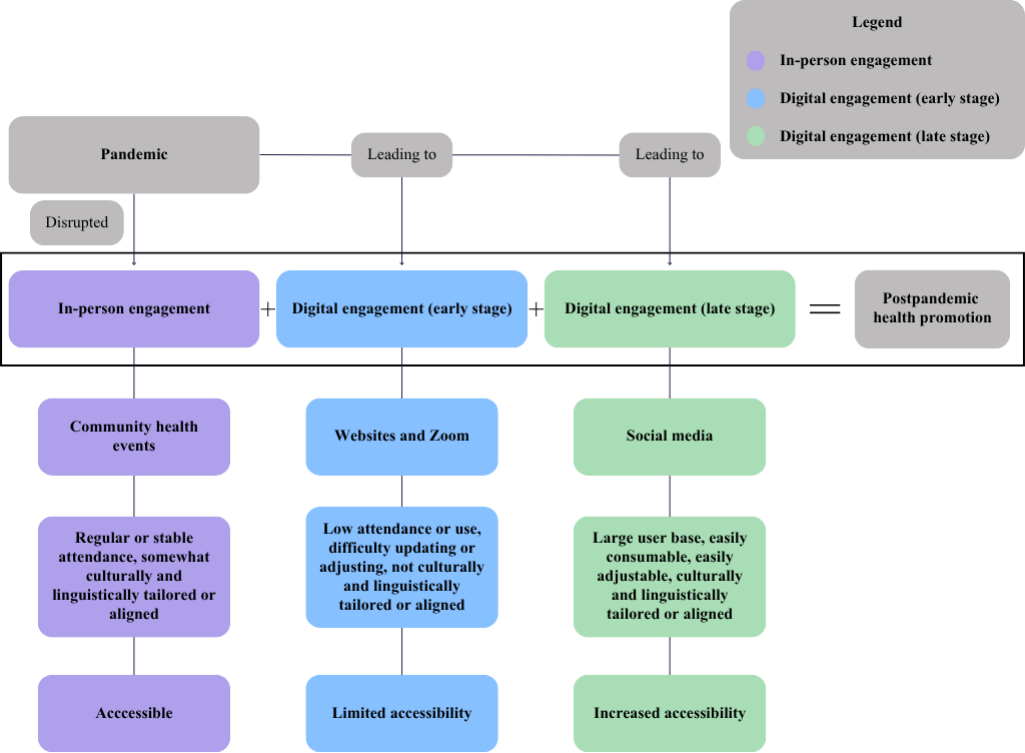
Strategies to promote brain health and ADRD awareness before, during, and after COVID-19. The flowchart illustrates the evolution of the ELHA Lab’s brain health and ADRD outreach in Latine and Hispanic communities in Los Angeles across 3 phases: pre–COVID-19 in-person events, COVID-19 era digital engagement, and post–COVID-19 adaptation. Initially, stable, accessible community events shifted to limited online engagement (early stage) with low accessibility and cultural misalignment. In response, ELHA developed a culturally tailored social media–based approach (late stage) using stakeholder insights, improving accessibility and engagement in a digital landscape. After COVID-19, it is suggested that effective brain health and ADRD prevention education requires a combined response using strategies from in-person engagement, early-stage digital engagement, and late-stage digital engagement. ADRD: Alzheimer disease and related dementia; EHLA: Equity for Latinx-Hispanic Healthy Aging.

The Equity for Latinx-Hispanic Healthy Aging (ELHA) Lab, at University of California, Los Angeles’ Department of Neurology [67] in Los Angeles County, launched the Latine-Hispanic Digital Brain Health Program in October 2023. Our objective was to sustain engagement with the US Latine and Hispanic communities in line with digital shifts in a postpandemic climate (Figure 1). We constructed the program by following the 5 key steps of design thinking, in line with human-centered design (HCD) approaches [68-70].

#### Empathizing With Stakeholders

From February 2022 to April 2023, ELHA staff conducted 29 informal phone and Zoom interviews with 27 Latine and Hispanic stakeholders, representing 14 Los Angeles–based community-based organizations that provide ADRD support services and resources and 2 Los Angeles–based ADRD caregivers. We collected information on the stakeholders’ involvement with and knowledge of community members’ experiences with prevention services (eg, physical health education and brain health education), dementia early detection services, diagnostic processes, dementia care planning and management, and ADRD research involvement. Additionally, stakeholders were prompted to share their perceptions of how systemic barriers and SDOH factors prior to and during the pandemic impacted accessing or delivering ADRD services.

#### Defining the Problem

Stakeholders specifically noted that the pandemic disrupted their organizations’ ability to communicate about brain health and ADRD prevention. This led to limited dissemination of brain anatomy education, current research information, and available health care and community resources communicated in line with the US Latine and Hispanic communities’ languages, cultures, needs, values, and traditions.

#### Generating Ideas for Solutions

**Figure 2.**
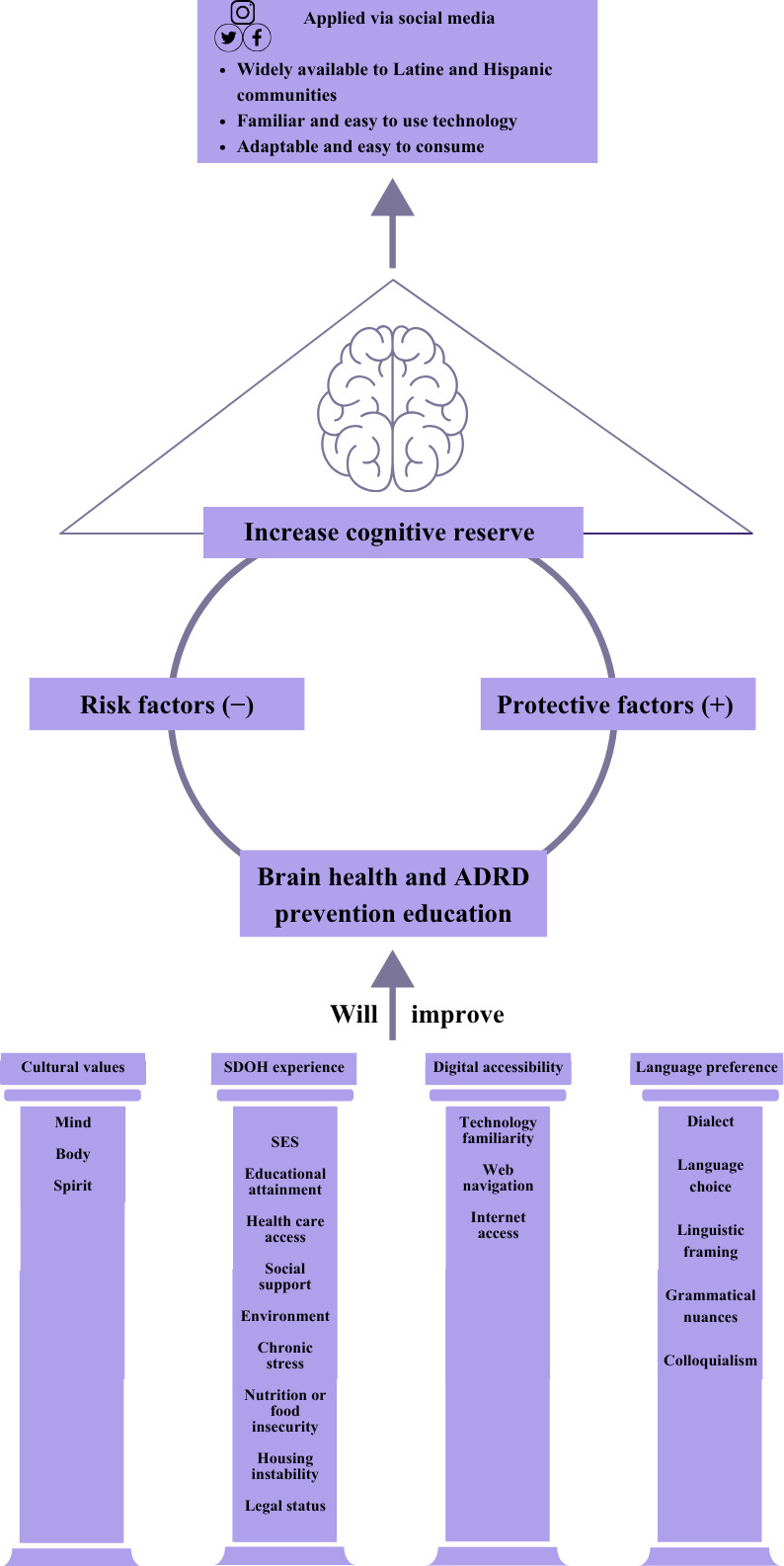
Digital Alzheimer Health Education Model. The Digital Alzheimer Health Education Model addresses unique SDOH, digital access, language, and cultural values (mind, body, and spirit), making brain health and ADRD information more accessible and relevant to the Latine and Hispanic cultures. In Latine and Hispanic cultures, health is addressed from a holistic perspective. The values of mind, body, and spirit reflect the cultural approach to taking agency over one’s health (eg, prioritizing mental well-being of the mind [la mente], respecting the body [el cuerpo] by providing it with healthy cultural food and engaging in community-rooted physical exercise, and feeding the spirit [el spiritu] through connecting with family, community, and faith) [92-94]. By tailoring content and engagement strategies to the Latine and Hispanic population’s definition of health, the model aims to overcome barriers to equitable brain health education and encourage participation in brain-healthy activities known to prevent ADRD [92]. ADRD: Alzheimer disease and related dementia; SDOH: social or structural determinants of health; SES: social economic status.

After defining these barriers, our team proceeded with creating an action plan to respond to stakeholders’ reported needs. In early 2023, the ELHA team and director held multiple brainstorming meetings to propose potential platforms and education approaches, resulting in the development of our brain health and ADRD prevention social media strategy. We considered both deep structures (ie, the underlying cultural, social, and systemic factors influencing health behaviors) and surface structures (ie, visual and linguistic alignment with the target community’s cultural identity) prior to the program’s development [71]. In response to stakeholder requests for culturally and linguistically responsive brain health and ADRD education or resources, the Digital Alzheimer Health Education Model (Figure 2) was created as a deep structure, theory-driven conceptual model for our program. Our model proposes potential pathways for enhancing cognitive reserve [13,48-57] by increasing collective knowledge on modifying risk factors for ADRD while addressing the unique barriers and facilitators that enable equitable access to brain health education (eg, information about keeping brain healthy through daily habits) and ADRD prevention resources (eg, tools, programs, and services to support ADRD prevention) for US Latine and Hispanic communities. In this model, we hypothesize that improving cognitive reserve relies on increasing brain health literacy and encouraging behavior changes that promote participation in brain-healthy activities while reducing risk behaviors and knowing the available community or health care resources in the local area in English and Spanish. We combined cognitive reserve theory with culturally and linguistically congruent health communication methods while considering the interplay of SDOH experiences, digital access or web literacy, language, and cultural values throughout the program [19,20,72-74].

Consistent with the social marketing and health communication theory described by Resnicow et al [71], we also recognized that a strong, culturally resonant brand using surface structure principles, like imagery and symbolism, enhances engagement and message retention through health branding. Our team had already begun the process of developing a consistent and recognizable brand for in-person community engagement with our logo and use of purple. This was further enhanced, as we developed our digital branding kit (a comprehensive guide that outlines the visual and verbal components of a brand) [75]. The ELHA Lab’s brand kit consists of our logo, color palette, slogan, and values statement, designed to reflect the cultural values and lived experiences of US Latine and Hispanic communities. The ELHA logo (Multimedia Appendix 1) comprises a butterfly and brain, symbolizing transformation, migration, and ancestral presence for brain health promotion [76-79]. Paired with our slogan, “Transformando Mente, Cuerpo y Espíritu en Comunidad para un Cerebro Sano [Transforming Mind, Body, and Spirit in Community for a Healthier Brain],” our brand symbolizes the communities’ resilience and adaptability on their journey toward a healthier brain. The purple color palette (Multimedia Appendix 2), aligning with the international color for Alzheimer, represents passion and stability in the fight against the disease [80]. These aspects of our health branding and our 3 pillars of social responsibility (ethical duty to improve society) [28,81-83], critical consciousness (awareness of oppression and action) [84-88], and language justice (equal access to all languages) [89-91] form the foundation of the program’s interface (eg, platform type, page esthetic, and content delivery), content (eg, topics, themes, and visuals), and engagement strategies (eg, reposts and comment responses). By integrating both deep and surface structure principles into our model and health branding, we address critical gaps identified by stakeholders and community members, namely, the lack of accessible brain anatomy education, up-to-date research information, and culturally and linguistically aligned health care resources. This approach ensures that our brain health education is culturally resonant, accessible, and drives meaningful community education, guided by continuous feedback from community stakeholders.

#### Prototyping the Solutions

Initial brain health and ADRD primary prevention educational content was created following the model using Canva (a graphic design platform; Canva Pty Ltd), and disseminated via 3 social media platforms, Facebook [95], Instagram [96], and X [97]. Initial community online feedback from Facebook and Instagram comments and live informal feedback from community members in ELHA’s social networks over the first month informed program development and content or strategy adjustments. No formal analysis was conducted on the initial feedback, but repeated themes and tailoring requests were being voiced. The team identified 5 of these recurring themes that aligned with previously voiced needs from initial stakeholder interviews and aspects of the model and developed evaluation guidelines for program adherence and content appropriateness, including consistency and rapport, cultural relevance, visual representation, linguistic congruency, and storytelling. These guidelines became concrete measures for content creation and dissemination to ensure that we were meeting the needs and preferences of community stakeholders and members.

#### Testing the solutions

During the prototyping phase, we deployed our digital program, incorporating the feedback and input collected from community members and stakeholders. Throughout the testing phase, comments from social media content and from community stakeholders and members of the team interacted with during in-person community engagement events informed slight adjustments in content creation and delivery. When noted, this feedback was presented at weekly meetings, and the team would collectively decide when an adjustment was to occur.

### Study Design or Program Guidelines

#### Content Management and Operations

##### Scheduling

Consistency and rapport are crucial when connecting with historically underserved communities [98]. Our team established an operational strategy to maintain a continuous digital presence. This began with regularly posting content on Mondays, Wednesdays, and Fridays at 11 AM [99,100]. Building credibility requires reliability, which in turn fosters the communities’ trust in the research laboratory [101,102].

As our audience and program grew, so did the need for strategy adjustment. To address community preferences, we introduced stories (ephemeral content on Facebook and Instagram) on Tuesdays and Thursdays [103]. Their effectiveness in maintaining visibility with minimal effort encouraged our expansion to 4‐10 stories daily, Monday to Friday [104]. To maintain engagement, we ensured timely content delivery with tools like Pallyy (Pallyy PTY LTD), a social media scheduling software.

##### Defining Themes and Narratives

Monthly content calendars, developed using Canva, were used to guide our strategy, ensuring that posts address cultural, brain health, and ADRD SDOH-related topics. The ELHA team proposed themes and potential content for each month at least 1‐2 weeks prior, and the ELHA director reviewed and approved the calendar before content was developed and disseminated. Monthly themes are aligned with noteworthy events and observations, such as Alzheimer’s and Brain Awareness Month in June (Multimedia Appendix 3), to optimize relevance and engagement. For example, during Caregiver Appreciation Month in November (Multimedia Appendix 4), content included self-care tips [105], family-centered (familismo) [106-109] resources, and insights into ADRD biology.

##### Content Creation Team

Having a team of 11 bilingual and bicultural Latine and Hispanic women students, staff, and faculty further supported program sustainability. Our diverse academic backgrounds in cognitive science, neuroscience, and related fields enriched content development while maintaining cultural and linguistic relevance through the lens of individual and collective intersectionality and positionality [110-115].

### Content Strategy

Our content strategy integrates storytelling, cultural relevance, linguistic inclusivity, and visual representation to create content that resonates with US Latine and Hispanic communities.

#### Storytelling and Cultural Relevance

As a culturally informed communication strategy, storytelling methods are used in posts and captions [64,116,117]. An example of this in our content can be observed in Multimedia Appendix 5. Multimedia Appendix 6 further exemplifies our use of culturally resonant narratives and values. ELHA uses these methods to connect the story and information to the real-life experiences and general knowledge of the US Latine and Hispanic communities [71,118].

#### Linguistic Inclusivity and Visual Representation

All content is presented in Spanish and English. Linguistic inclusivity necessitates nuanced approaches to selecting the right words, phrasings, metaphors, and syntax. Recognizing the diversity in education and language within the US Latine and Hispanic communities, ELHA diligently works to use simple language [118]. This is achieved by using translation software like DeepL (DeepL SE) for initial translations, followed by a review and editing process by the ELHA team, comprising Spanish-English bilinguals with various backgrounds. Additionally, ELHA’s director (a native Spanish speaker) reviews, edits, and approves all posts.

Visual representation is equally crucial in fostering inclusion [119,120]. We carefully select or create imagery that reflects the diversity of the communities, considering skin tone, hair texture, and other characteristics (Multimedia Appendix 7). Our approach aims to make community members feel seen and valued, reinforcing their connection to the content. We source and create representative images on platforms like Canva and FreePik (FreePik Company SL).

#### Iterative Adaptations

In line with principles of HCD for adaptation, we follow an informal iterative approach to our content and strategy rather than through a defined protocol. This process occurs through periodically observing comments and gathering feedback from community events the team engaged in. Then, team members would bring up feedback at weekly lab meetings as they were presented and discuss strategies that could improve the social media based on feedback and affirming trends in social media analytics. Examples of these changes include allotting more resources to engage with other pages or groups on Facebook (started June 2024); adding blogs in English on topics related to brain health, ADRD prevention, and Latine and Hispanic culture to address community requests for more information (started April 2024); and including more video-based content (throughout 2024), contributing to the cultural relevance of the program. All of this is done in response to informal feedback received by live community members and stakeholders in ELHA’s social network and on Facebook and Instagram comments.

### Data Collection and Analysis

#### Overview

A convergent mixed methods analysis was conducted. Our team simultaneously analyzed quantitative results from social media analytics and qualitative results from social media comments. We then compared or contrasted the results from both analyses.

#### Quantitative Metrics

We used Meta Business Suite Analytics [121] for quantitative data, analyzing key metrics of social media engagement for the first 14 months after implementation (October 2023 to December 2024), including:

1. Follows: Tracks growth by the number of individuals subscribing to our social media
2. Unfollows: Tracks the number of individuals unsubscribing from our social media
3. Followers: The current number of subscribers to our social media
4. Reach: Tracks the number of unique individuals who have viewed our content
5. Impressions: Tracks the number of times our content is viewed
6. Interactions: Tracks engagement through actions, such as likes, comments, and shares

The analysis focused on using descriptive statistics to evaluate trends in program growth, reach, and engagement. We collected and analyzed data from Facebook and Instagram only. X analytics was excluded due to data access limitations. For X, followers are the only reported measure available. Note, no paid advertisements or additional paid boosts were used, so the data presented are findings for organic posts (content shared without paid promotion) only. Findings are presented based on content type (Table 1).

**Table 1.** Content types definitions^a^.

Content type	Definition
Post	Any piece of content shared on social media.
Repost	Content from other creators that is reshared onto our platform.
Text	A written post with no image or video.
Photo or image	A single visual, such as a photo, graphic, or flyer.
Carousel	A swipeable post with multiple photos, graphics, or flyers.
Video or reel	A short, vertical video.
Story	A post that disappears after 24 hours.

a The table defines common terminology used to describe social media content.

#### Qualitative Analysis

We compiled social media comments (Multimedia Appendix 8) available on Meta Business Suite Analytics (comments from Facebook and Instagram posts) as well as 2 verbal comments recorded from caregivers who have explored our social media. Comments from Instagram stories and X in general were unavailable. A combined digital and manual sentiment analysis was then conducted to evaluate perceptions of the program [122-125]. Our team developed a codebook to define positive, negative, and neutral sentiment, iterating upon previous literature (Multimedia Appendix 9) [125-129]. Two coders (AM and SO-E) used the agreed-upon codebook to conduct the analysis independently and then collectively met with the principal investigator (MD-S) to resolve disputes. A Cohen κ analysis was conducted to measure interrater reliability of the individual responses from coder 1 (AM) and coder 2 (SO-E). A software-coded sentiment analysis was then conducted using the artificial intelligence (AI)–assist, ant ChatGPT, for comparison. Our team began by uploading the codebook to ChatGPT and prompting, “retain the information provided in the codebook.” Once this was complete, we asked ChatGPT to conduct a sentiment analysis by uploading all the comments and prompting, “Based on the codebook, provide your interpretation of sentiment for each of the following comments.”

### Ethical Considerations

This study was not submitted for institutional review board review due to the use of publicly available information only. All presented information (eg, comments) has been deidentified to ensure the safety and security of commenters. Upon conversations with institutional review board administrators, this study was deemed not human subjects research as defined by the federal regulations 45 CFR 46 [130].

## Results

### Quantitative Metrics

#### Content

In total, 2440 pieces of content were created or reposted, including 368 Facebook posts and reposts, 366 Instagram posts, and 1706 Instagram Story posts and reposts.

#### Followers or Follows

**Figure 3.**
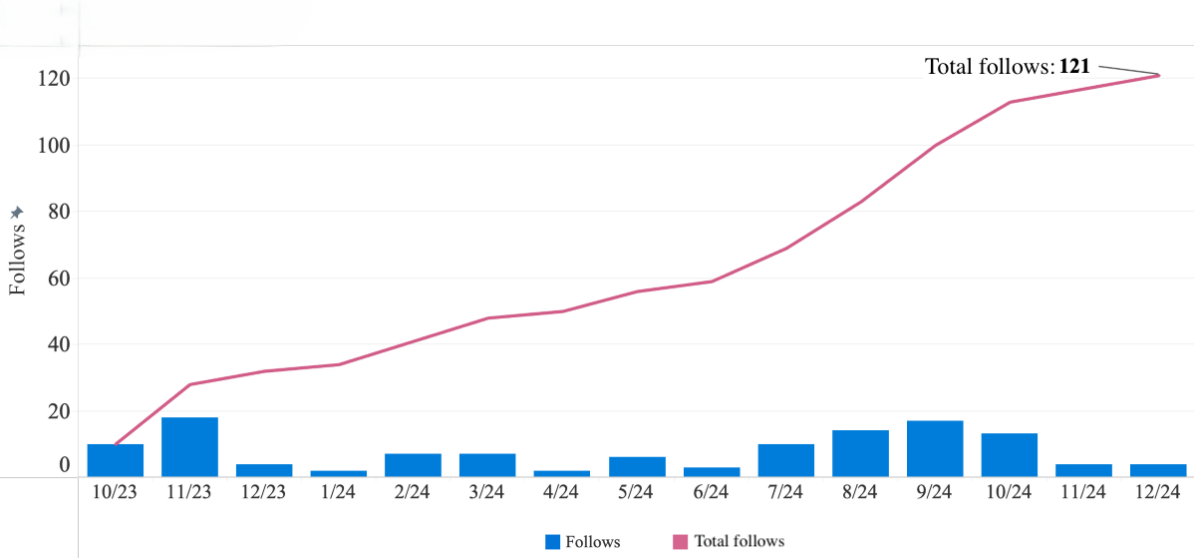
Latine-Hispanic Digital Brain Health Program’s Facebook follows. The figure depicts the program’s total Facebook follows (n=121) from October 2023 to December 2024, as well as the follows per each calendar month.

**Figure 4.**
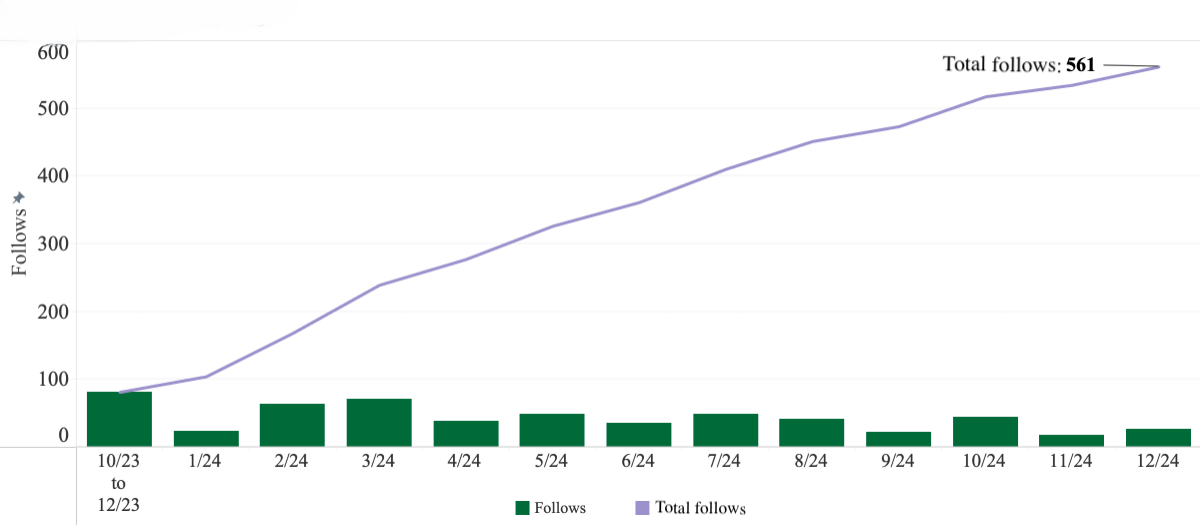
Latine-Hispanic Digital Brain Health Program’s Instagram follows. The figure depicts the program’s total Instagram follows (n=561) from October 2023 to December 2024, as well as the follows per each calendar month. Due to limitations on data availability, months 1-3 had to be aggregated into 1 column.

As of December 31, 2024, a total of 857 followers were accrued across all our social media platforms (Instagram: n=534; Facebook: n=124; and X: n=199). We gained 121 follows on Facebook (Figure 3) and 561 follows on Instagram (Figure 4). There were no recorded unfollows on Facebook, and we recorded 27 unfollows on Instagram. Note, 3 followers consisting of ELHA team members were subscribed to our Facebook platform before the observed analysis period and were not accounted for in the “follows” category.

#### Facebook Reach and Engagement

On Facebook, we reached a total of 20,904 individuals across the platform from October 2023 to December 2024. Table 2 describes the reach, interactions, and engagement during the observation period. On average, photo or image posts had the highest reach, impressions, and interactions. This was followed by carousel posts with the second highest reach and impressions, and videos or reels with the second highest interactions.

A total of 2323 interactions were recorded, consisting of 1531 (65.91%) reactions, 765 (32.93%) shares, and 27 (1.16%) comments. There was an observed preference for reactions (likes and emojis), followed by shares, and comments (Multimedia Appendix 10).

**Table 2.** Latine-Hispanic Digital Brain Health Program’s Facebook reach, impressions, and interactions^a^.

Content type	Reach, mean (SD)	Impressions, mean (SD)	Interactions, mean (SD)	Content, n (%)
Carousel	60.79 (88.88)	68.69 (95.61)	5.68 (8.34)	245 (66.58)
Photo or image	103.43 (205.95)	117.43 (235.25)	9.86 (11.45)	7 (1.96)
Text	12.43 (9.57)	15.57 (11.72)	0.43 (0.79)	7 (1.96)
Video or reel	47.71 (71.65)	52.81 (78.35)	7.89 (14.56)	109 (29.62)
All content	56.80 (87.02)	63.90 (94.56)	6.31 (10.62)	368 (100)

a This table depicts average reach, impressions, and interactions across Facebook content types (carousels, photo or image posts, videos or reels, and stories) published by Equity for Latinx-Hispanic Healthy Aging from October 2023 to December 2024 (n=368).

#### Instagram Reach and Engagement

On Instagram, we reached a total of 136,564 (posts: n=57,759 and stories: n=78,805) individuals across the platform from October 2023 to December 2024. Table 3 describes the reach, interactions, and engagement during the observation period. On average, video or reel content had the highest reach and impressions, followed by photo or image posts. Photo or image posts had the highest average interactions, followed by videos or reels.

Instagram post interactions totaled 6082, consisting of 5104 (83.92%) reactions, 583 (9.59%) shares, 122 (2.01%) comments, and 273 (4.49%) saves. Most interactions were reactions, followed by shares, saves, and comments (Multimedia Appendix 11). Stories yielded 3004 interactions, including 1828 (60.85%) likes, 382 (12.72%) profile visits, 507 (16.88%) sticker taps, 92 (3.06%) link clicks, 161 (5.36%) replies, and 34 (1.13%) shares, with the majority being likes and sticker taps (emoji responses; Multimedia Appendix 12).

**Table 3.** Latine-Hispanic Digital Brain Health Program’s Instagram reach, impressions, and interactions^a^.

Content type	Reach, mean (SD)	Impressions, mean (SD)	Interactions, mean (SD)	Content, n (%)
Carousel	96.67 (74.26)	165.67 (128.50)	14.30 (12.73)	229 (11.11)
Photo or image	246.71 (136.10)	376.71 (241.47)	29.14 (13.17)	7 (0.34)
Video or reel	285.63 (358.39)	391.61 (465.37)	21.69 (13.85)	120 (6.06)
Story	46.19 (290.12)	47.44 (294.04)	1.76 (2.36)	1706 (82.74)
All content	66.22 (284.69)	81.53 (305.94)	4.41 (8.38)	2062 (100)

a This table depicts average reach, impressions, and interactions across Instagram content types (carousels, photo or image posts, videos or reels, and stories) published by Equity for Latinx-Hispanic Healthy Aging from October 2023 to December 2024 (n=2062).

### Qualitative Analysis

We evaluated 130 of 151 comments in the sentiment analysis. In total, 21 comments were excluded, as 18 originated from the ELHA team, and 2 were marked unavailable. Our sentiment analysis revealed overall positive sentiment, with 117 (90%) of the comments classified as positive in the software-coded analysis, and 113 (86.92%) in the hand-coded analysis. In both analyses, no negative sentiment was identified. A smaller proportion of the content was classified as neutral, with 8 (6.15%) in the software-coded analysis, and 10 (7.69%) in the hand-coded analysis. Additionally, some comments were deemed irrelevant, accounting for 5 (3.85%) in the software-coded analysis, and 7 (5.39%) in the hand-coded analysis (Table 4). Our Cohen κ analysis revealed substantial agreement between raters (κ=0.70; SE=0.09; 95% CI 0.52-0.88; *P*<.001).

**Table 4.** Sentiment distribution of Latine-Hispanic Digital Brain Health Program’s social media comments^a^.

Sentiment	Software coded (ChatGPT), n (%)	Hand coded, n (%)	Comment examples
Positive	117 (90)	113 (86.92)	“It always makes me so excited to see multilingual resources in dementia! Great work friend! ��” “Excellent conversation and advocacy for the Latino/a/e community ❤️ Buena charla. Gracias por todo su abogacía para la comunidad [Good talk. Thank you for all your advocacy for the community.] ��🏽”
Negative	0 (0)	0 (0)	N/A^b^
Neutral	8 (6.15)	10 (7.69)	“Muy interesante como abordar el trauma con las personas con alguna demencia. @mirella.diaz deberia de invitar la redcatedusername al grupo [Very interesting how to address trauma with people with dementia. @mirella.diaz should invite redcatedusername to the group.] 🙌🙌”
Irrelevant	5 (3.85)	7 (5.39)	“D’m_me_pls> @redactedusername”
Excluded	21 (N/A)	21 (N/A)	“Gracias a usted por estar conmigo hoy ❤️ [Thank you for being with me today.] [ELHA Response]”
Total	151 (100)	151 (100)	N/A

a This table summarizes both software-assisted (ChatGPT) and independent hand-coded qualitative sentiment analysis of 151 Instagram and Facebook comments posted in response to Equity for Latinx-Hispanic Healthy Aging’s bilingual, community-engaged brain health and Alzheimer disease and related dementia education content targeting Latine and Hispanic audiences.

b N/A: not applicable.

## Discussion

### Principal Findings

The purpose of this study was (1) to describe the design and pilot study of our digital brain health and ADRD prevention program for English and Spanish-speaking, US Latine and Hispanic communities, using 3 social media platforms (Facebook, Instagram, and X) and (2) to present the quantitative and qualitative results from the first 14 months. The program implements principles from our model, which integrates 3 evidence-based frameworks of ADRD (cognitive reserve theory [13,48-57], SDOH [7,58-62] in ADRD, and human aging neuroscience), with deep structures (ie, the underlying cultural, social, and systemic factors influencing health behaviors) and surface structures (ie, visual and linguistic alignment with the target community’s cultural identity) principles to align with the needs, values, preferences, and traditions of US Latine and Hispanic communities [71]. In response to the feedback from stakeholders regarding a lack of brain anatomy education, current research information, and available health care and community resources communicated in line with the US Latine and Hispanic communities’ languages, cultures, needs, values, and traditions, our team created an iterative, community-informed process. The model incorporates culturally resonant storytelling, bilingual content, visual branding, and social media strategies to deliver accessible brain health and ADRD prevention education while continuously adapting based on stakeholder feedback.

Our quantitative analysis revealed consistent engagement throughout the evaluation period across Facebook and Instagram, with an increase in following: expansive reach, broad impressions, and consistent community interactions. On Facebook, photo or image posts had the highest interactions (mean 9.86, SD 11.45) and the greatest average reach (mean 103.43, SD 205.95), followed by video or reels with 7.89 (SD 14.56) interactions and an average reach of 47.71 (SD 71.65; third after carousels). On Instagram, video or reels had the highest average reach (mean 285.63, SD 358.39), while photo or image posts led in interactions (mean 29.14, SD 13.17), with an average reach of 246.71 (SD 136.10). Due to data access unavailability from X, we could not present these results. Qualitative sentiment analysis from Facebook and Instagram showed that content was mostly perceived positively, with no negative sentiments. Cohen κ analysis revealed substantial agreement between raters.

### The Digital Divide and Social Media as a Digital Health Tool: A Tailored Approach

The COVID-19 pandemic rapidly accelerated the transition to digital health services, highlighting and exacerbating existing disparities in digital access (ie, digital divide) among US Latine and Hispanic communities [28-33]. While many early ADRD education and prevention programs relied heavily on websites and Zoom, the digital shift inadvertently excluded individuals with limited digital literacy, unreliable internet access, and/or technological barriers, as shown by the lack of US Latine and Hispanic engagement in a study by Gutiérrez et al [30] on website-based ADRD education workshops. As technology continues to serve as a central form of engagement in a post–COVID-19 climate, alternative platforms needed to be explored to surmount digital divide barriers [27]. Our findings emphasize that social media platforms may be an effective avenue to engage Latine and Hispanic individuals when content is tailored for their priorities and needs. This directly addresses stakeholder comments from our initial informal interviews, which revealed that there were a lack of available information and resources regarding brain health and ADRD prevention available in Spanish and in line with Latine and Hispanic cultural values. While we observed quantitative expansiveness in reach and engagement, showing accessibility to large communities, qualitative community feedback highlighted the importance of representation and language accessibility in fostering meaningful engagement.

Early in the COVID-19 pandemic, major ADRD organizations, such as Alzheimer’s Association and Alzheimer’s Los Angeles, revamped their websites to ensure equitable access to digital resources, including adding varied languages (through Google translation tools), updating their resource pages (limited availability in Spanish), and remodeling website aesthetics to be more user-friendly [27]. As necessary as these changes were, US Latine and Hispanic individuals continued to limitedly engage in digital health education, as they still faced barriers with deep and surface-level factors, such as language and cultural relevance [71,105,131]. For example, in the study by Rodriguez et al [131] looking at digital diabetes education, it was noted that people with limited English proficiency were less likely to engage in digital education and called for culturally tailored digital toolkits. Andrade et al [132], in contrast, demonstrated the effectiveness of a culturally and linguistically tailored digital community health worker model that disseminated COVID-19 information in Spanish using social media, significantly increasing engagement and knowledge retention among US Latine and Hispanic users. Similarly, our findings demonstrate that digital health education requires deep cultural and linguistic congruence to ensure feasibility and acceptability [3,133,134]. Qualitative comments to our digital program, such as “It always makes me so excited to see multilingual resources in dementia! Great work friend! 💜” in an Instagram and Facebook video or reel with a bilingual Latina social worker and “Amen! So common with Latinos. Keep educating *la gente* [the people]!” on a post on high blood pressure in the community and its effects on brain health, are examples of our content’s relevance and acceptability [73,135].

### The Power of Iteration and Cultural Responsiveness

Our findings suggest that the intervention, developed using evidence-based theories and piloted for content relevance and acceptability, effectively responds to the needs and preferences of the target audience, as reflected in the overwhelmingly positive qualitative feedback from community members. Our program used HCD approaches to establish a framework for initially creating and then continuously adapting the program’s format and content based on community needs and feedback to sustain engagement and relevance [68-70]. Göttgens and Oertelt-Prigione’s review of HCD in health research reinforces the value of iterative, user-driven approaches, demonstrating how HCD ensures that solutions remain adaptable and aligned with user needs throughout the innovation process. Consistently, the iterative and adaptive nature of our digital program, such as incorporating more video content and expanding storytelling formats, enhanced cultural responsiveness by meeting the community’s needs, preferences, values, and traditions. For instance, feedback, such as “I like the videos. For me, the videos are like experiences. When I see, I remember,” in conjunction with the substantial reach, impressions, and interactions of video-based content, informed the integration of more videos, reinforcing the importance of visually immersive learning [68,120]. The data also informed a shift from longer format carousel posts (5‐10 slides) to shorter format posts (2‐4 slides). We noted that there was higher reach and engagement of single photo or image posts on Facebook and higher engagement of these types of posts on Instagram. However, due to the bilingual format of the program, we are only able to use this format with simple posts (eg, holiday observations and team photos).

Additionally, expanding to blogs provided in-depth materials for those who prefer a longer reading format, and increasing Facebook engagement by connecting with other relevant community pages helped amplify outreach. These adaptations deepened community participation and strengthened connection to the program [68,136]. This iterative approach aligns with the work of Adam et al [68], who refined their video-based health education program for medical students by incorporating regular focus group feedback. Their adjustments to language, visual style, and messaging, based on participants’ preferences, ultimately enhanced audience engagement.

Our pilot study also adds to the current body of literature, emphasizing the importance of diverse and representative research teams in designing and implementing culturally and linguistically resonant health communication strategies [63,65,113-115]. Bains et al [137] demonstrated that teams with members who share cultural and linguistic similarities with the target population were more successful in designing communication strategies that resonated with community values, thereby increasing trust and participation. Similarly, Carter et al [138] highlighted how diverse research teams were better equipped to identify and address cultural nuances, ensuring that health messaging was both relevant and accessible. The lived experiences and cultural awareness of our team played a crucial role in fostering trust and ensuring that content is both relevant, impactful, and acceptable.

### Limitations and Future Directions

Our study has several limitations that shape both the overall design and the pilot findings. First, as is common in early formative research, there are methodological gaps. Due to financial and workforce constraints, we were unable to conduct multiple rounds of formal semistructured interviews or focus groups with community stakeholders throughout the development process, which would more fully align with HCD principles. Similarly, we were not able to implement a structured feedback and iteration process during the testing phase, limiting our ability to refine the program in real time. Another important limitation is the absence of a formal data-integration process in this pilot. Although mixed methods data were collected, systematic integration, consistent with a convergent mixed methods design, will be conducted in a subsequent, fully powered study.

A lack of culturally, racially, and linguistically representative images easily available for Latine and Hispanic communities is another limitation, requiring the team to manually adjust elements like hair type, skin tone, and language used in images, which can be time-intensive. Note, this study references a point in time before widespread use of AI. Since this period, rapid development in AI generation capabilities has improved the team’s ability to generate images that are culturally representative. Additionally, the absence of social media professionals on the team meant the program lacked strategic expertise that could have optimized engagement. Platform constraints such as character limits, video length limits, and technical issues (scheduling, platform outages, etc) created scheduling inefficiencies that also may have led to mistrust among the audience. The limited availability of demographic data from social media platforms is also a limitation. The platforms provide data on age, sex (male or female), use by city, and use by country. However, the unavailability of racial or ethnic demographics prevents a thorough assessment of Latine and Hispanic engagement and limits our ability to explore program use in these communities.

Another limitation was the unavailability of data on X, making it difficult to evaluate success on the platform. Due to an inability to evaluate engagement on this platform, in addition to a perceived mismatch in our approach with the platform’s target audience (only 22% of Latine and Hispanic individuals use it) [32], our team halted the use of X after the observed time period (October 2023 to December 2024). Future research should conceptualize new ways to tailor brain health and ADRD prevention education for X. For example, a member of our ELHA team is currently in the process of developing a new program to facilitate brain health and ADRD prevention education engagement with academics and policymakers who make up a large niche of the platform.

Future research should address these limitations by integrating professionals with expertise in digital engagement, leveraging AI-driven tools to streamline content creation, and implementing experimental designs to systematically assess community interactions, engagement, knowledge retention, and, ultimately, behavioral change. Collecting demographic data through in-platform surveys would allow for targeted evaluations of program effectiveness within and outside US Latine and Hispanic communities. Additionally, testing out paid posts or advertisements may also improve the visibility of posts and may be considered in the future. Furthermore, while our study did not measure behavioral change, future research will assess cognitive engagement through pre- and postintervention surveys and digital neuropsychological assessments to evaluate long-term impact. Moving forward, the insights from this study will serve as a foundation for developing and implementing culturally and linguistically responsive digital health education, with the next phase focusing on experimental methodologies to assess efficacy and effectiveness.

### Conclusions

US Latine and Hispanic communities experience a 1.5 times increased risk of developing ADRD compared to non-Hispanic White people and face disparities in ADRD detection, diagnosis, treatment, and care management [1,4-9,73]. The ELHA Latine-Hispanic Digital Brain Health Program demonstrates the potential of Facebook and Instagram as acceptable platforms for delivering brain health and ADRD primary prevention information when coupled with culturally and linguistically congruent strategies. ELHA’s approach provides an innovative methodological framework for researchers developing similar programs, demonstrating that culturally and linguistically congruent social media strategies paired with evidence-based ADRD research frameworks [7,13,48-55,57-59,61,62] and HCD [68-70] can engage and retain communities at higher risk of ADRD. Future public health initiatives should consider leveraging these methods to expand digital health education efforts for US Latine and Hispanic populations.

## Supplementary material

10.2196/73445 Multimedia Appendix 1 Equity for Latinx-Hispanic Healthy Aging logo.

10.2196/73445 Multimedia Appendix 2 Equity for Latinx-Hispanic Healthy Aging color palette.

10.2196/73445 Multimedia Appendix 3 Alzheimer’s and Brain Awareness Month sample calendar.

10.2196/73445 Multimedia Appendix 4 Caregiver Appreciation Month sample calendar.

10.2196/73445 Multimedia Appendix 5 You and Your Brain post example.

10.2196/73445 Multimedia Appendix 6 4 Healthy Habits for This Holiday Season post example.

10.2196/73445 Multimedia Appendix 7 Social media images and snapshots.

10.2196/73445 Multimedia Appendix 8 Social media comments.

10.2196/73445 Multimedia Appendix 9 Codebook for sentiment analysis of social media comments.

10.2196/73445 Multimedia Appendix 10 Latine-Hispanic Digital Brain Health Program’s Facebook interactions breakdown. The chart includes the total interactions (n=2323) for Facebook from October 2023 to December 2024, followed by the ways in which our audience is interacting with our content (eg, likes, shares, and reactions).

10.2196/73445 Multimedia Appendix 11 Latine-Hispanic Digital Brain Health Program’s Instagram post interactions breakdown. The chart includes the total interactions for Equity for Latinx-Hispanic Healthy Aging’s Instagram posts from October 2023 to December 2024 (n=6082) followed by the ways in which our audience is interacting with our content (eg, reactions, shares, and comments).

10.2196/73445 Multimedia Appendix 12 Latine-Hispanic Digital Brain Health Program’s Instagram story interactions breakdown. The chart includes the total interactions for Equity for Latinx-Hispanic Healthy Aging’s Instagram stories from October 2023 to December 2024 (n=3004) followed by the ways in which our audience is interacting with our content (eg, likes, shares, and replies).

## Abbreviations

ADRD: Alzheimer disease and related dementia
AI: artificial intelligence
ELHA: Equity for Latinx-Hispanic Healthy Aging
HCD: human-centered design
SDOH: social or structural determinants of health
