# Supplementary figures and images for: Methodological Framework for the Design and Implementation of a US Latine-Hispanic Digital Brain Health Program: User-Centered Design Approach
Source: JMIR Form Res. 2026 May 14;10:e73445. doi: 10.2196/73445 (PMC13175527; doi:10.2196/73445)

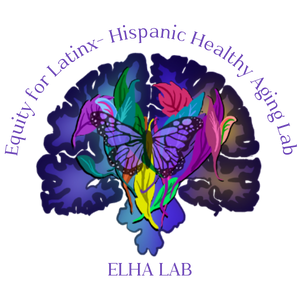

Supplement: Multimedia Appendix 1 [file formative-v10-e73445-s001.png]

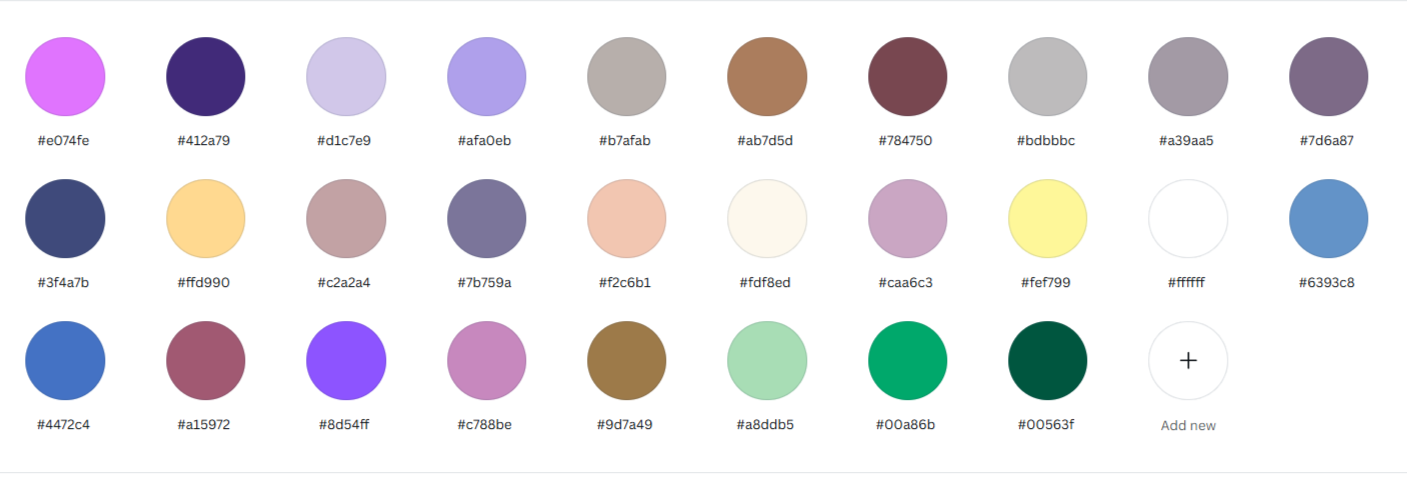

Supplement: Multimedia Appendix 2 [file formative-v10-e73445-s002.png]

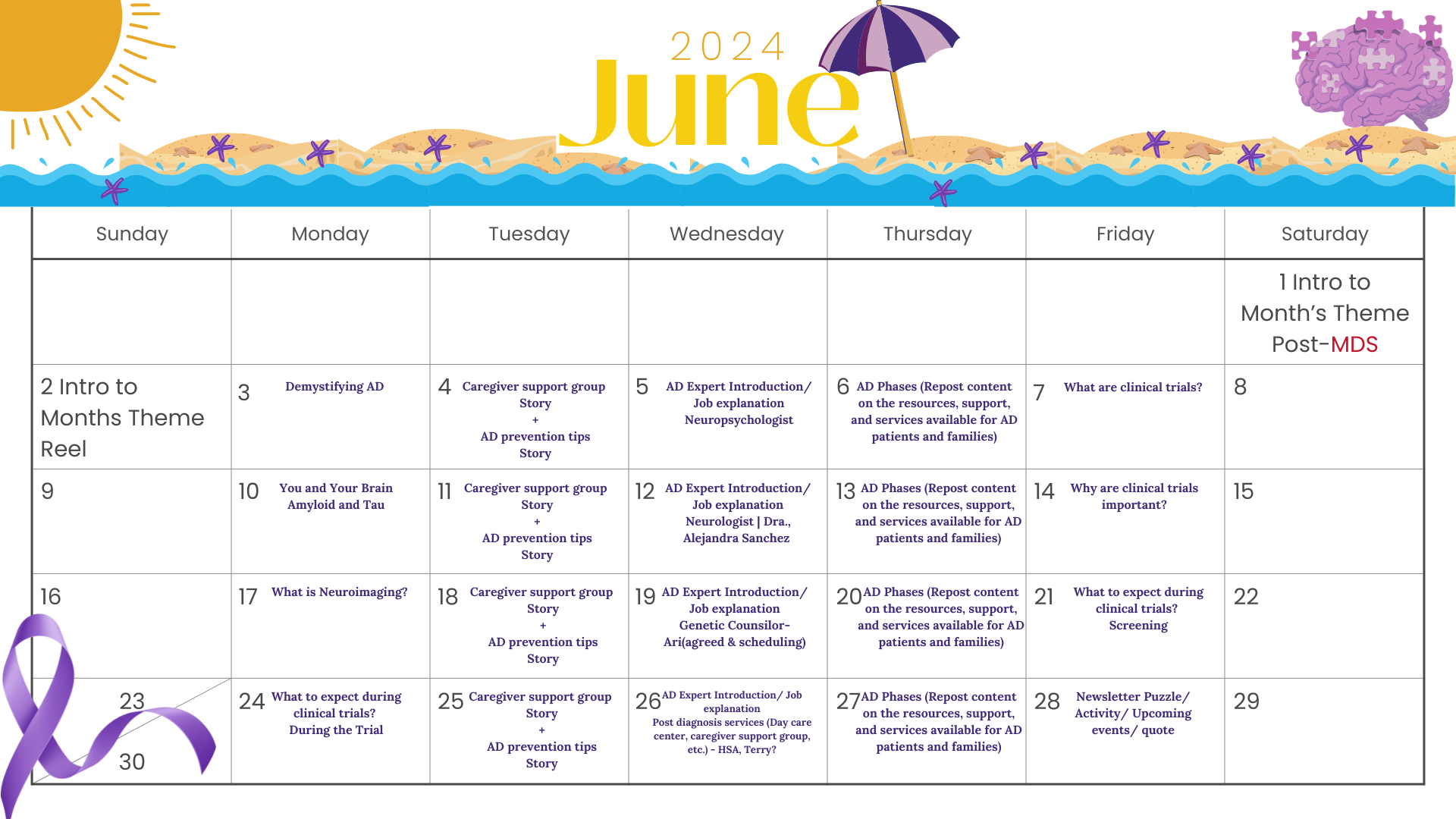

Supplement: Multimedia Appendix 3 [file formative-v10-e73445-s003.png]

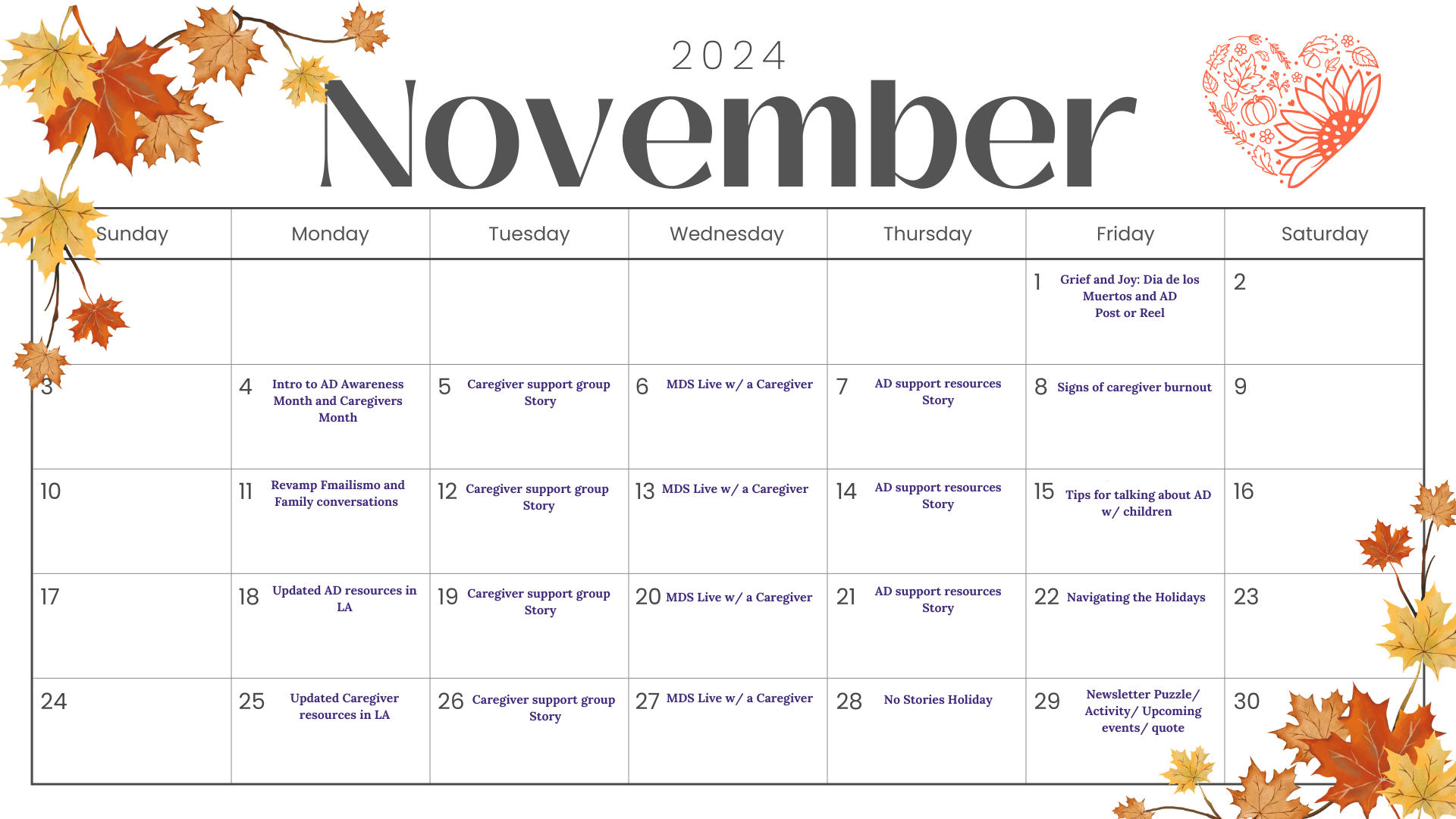

Supplement: Multimedia Appendix 4 [file formative-v10-e73445-s004.png]

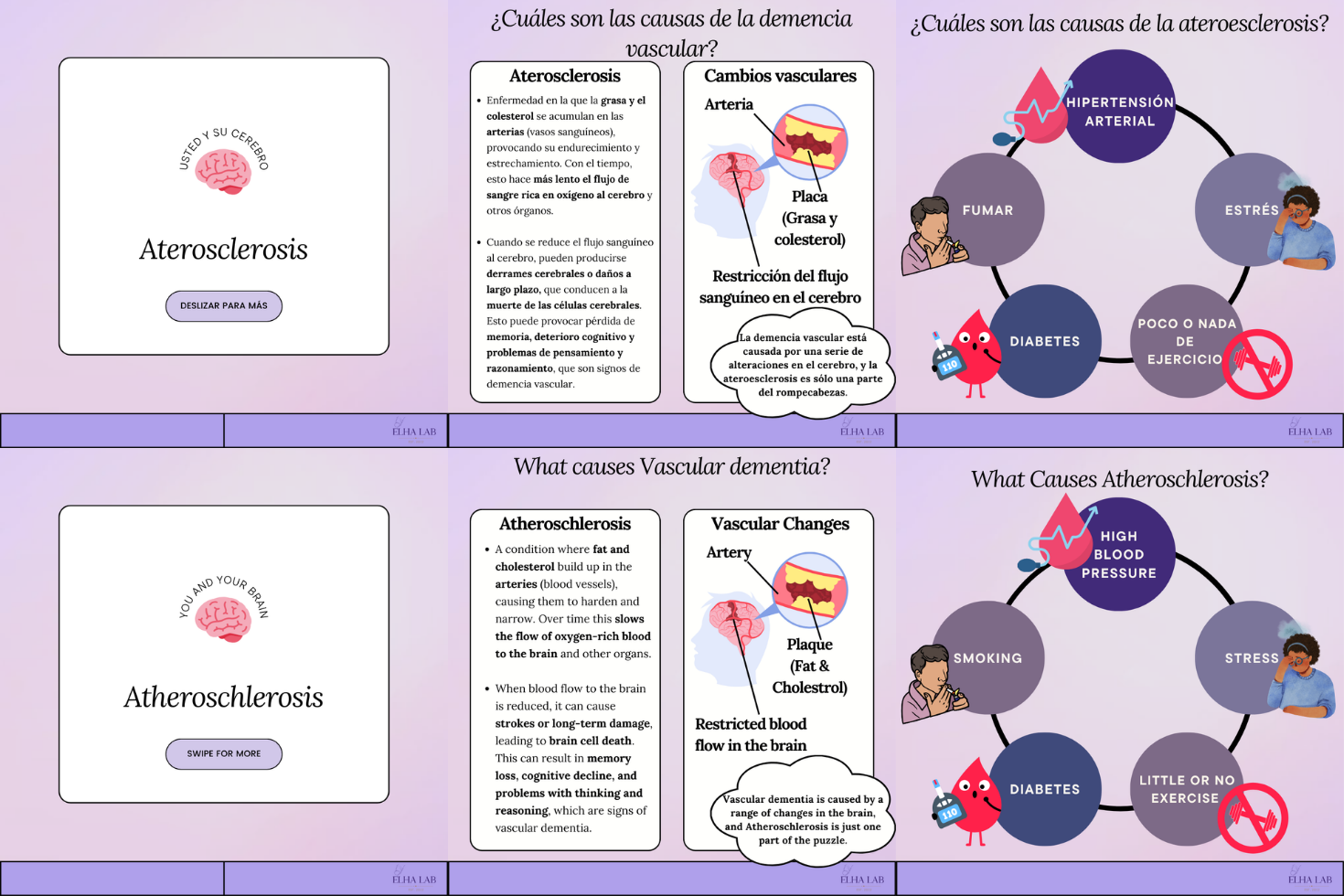

Supplement: Multimedia Appendix 5 [file formative-v10-e73445-s005.png]

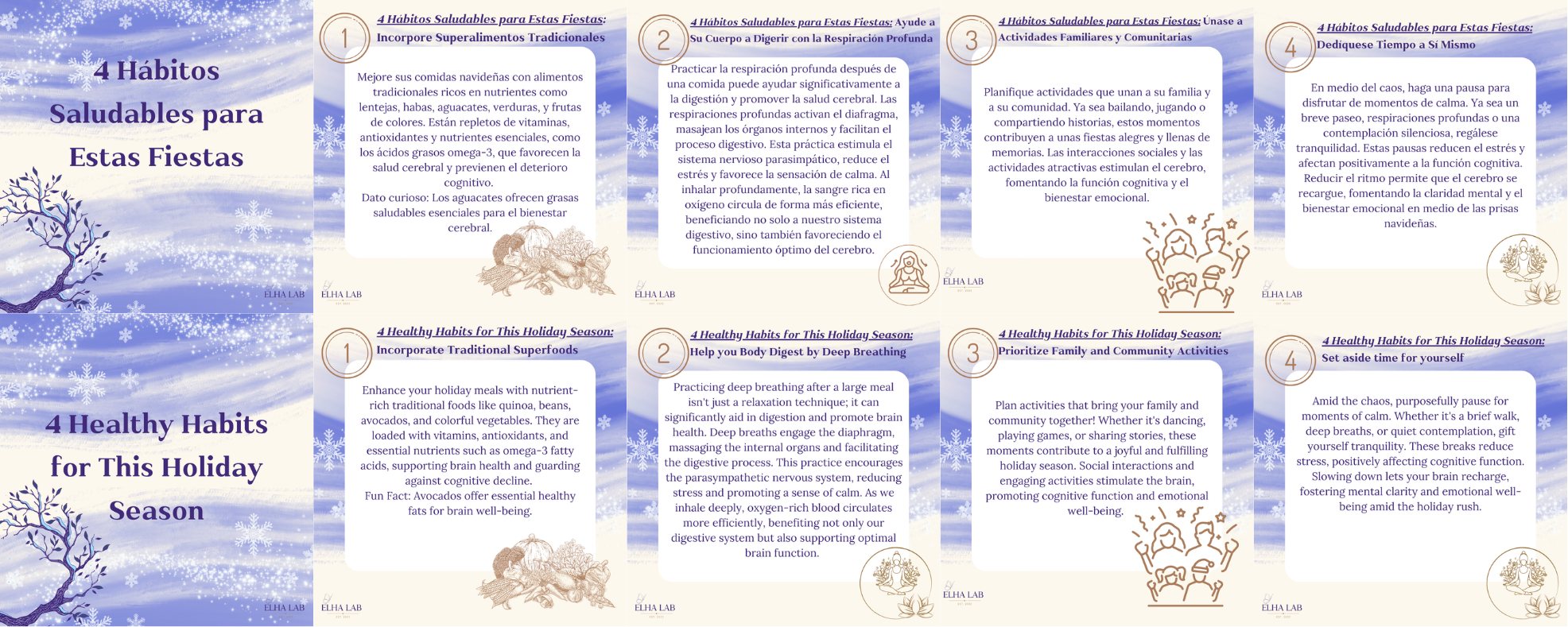

Supplement: Multimedia Appendix 6 [file formative-v10-e73445-s006.png]

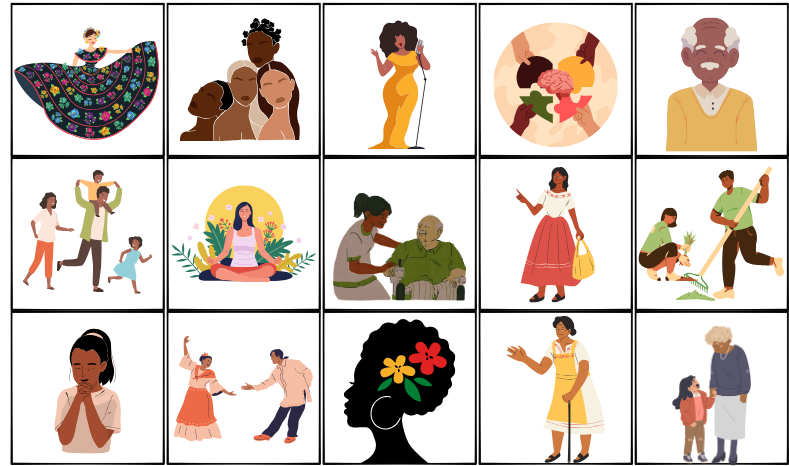

Supplement: Multimedia Appendix 7 [file formative-v10-e73445-s007.png]

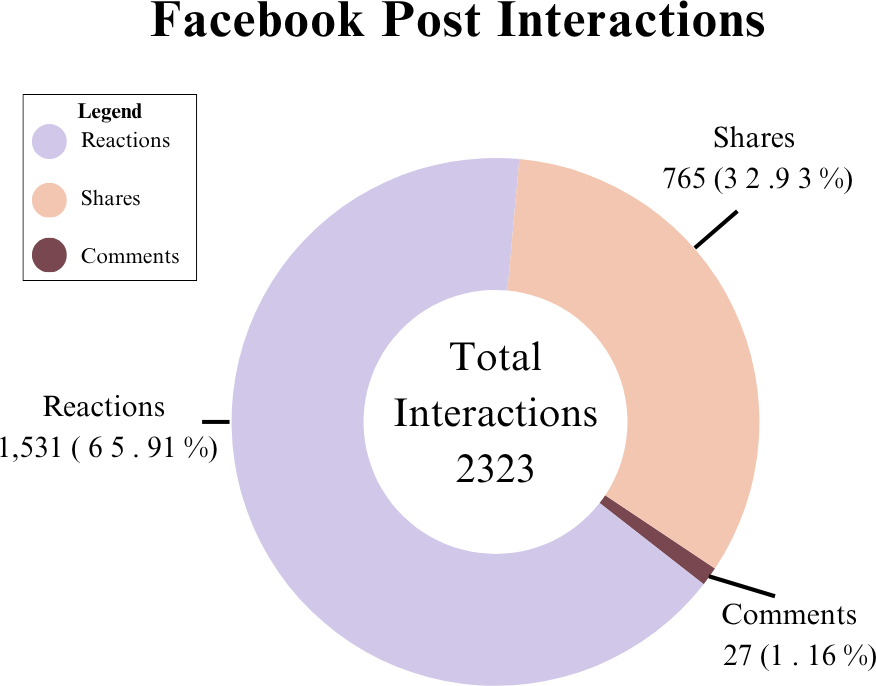

Supplement: Multimedia Appendix 10 [file formative-v10-e73445-s010.png]

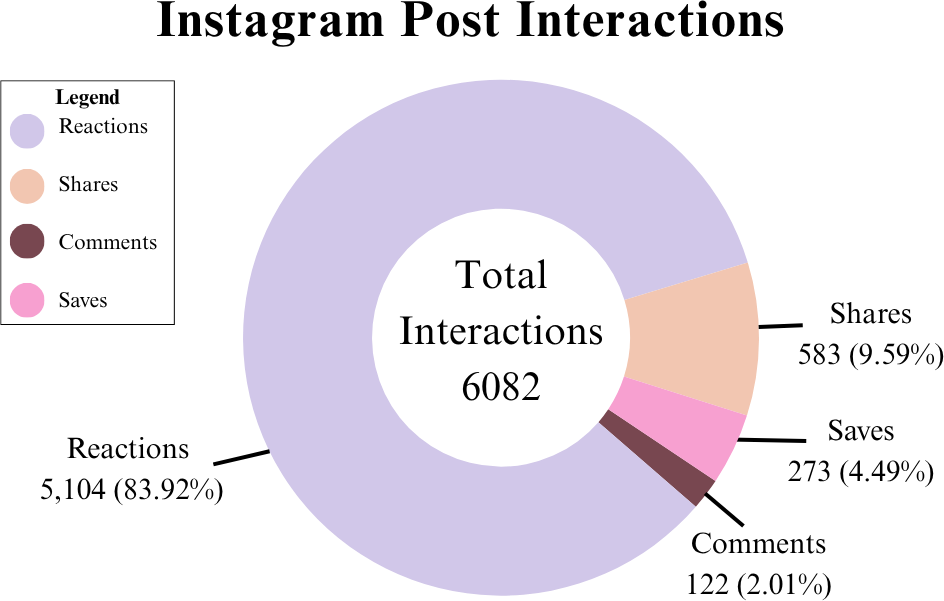

Supplement: Multimedia Appendix 11 [file formative-v10-e73445-s011.png]

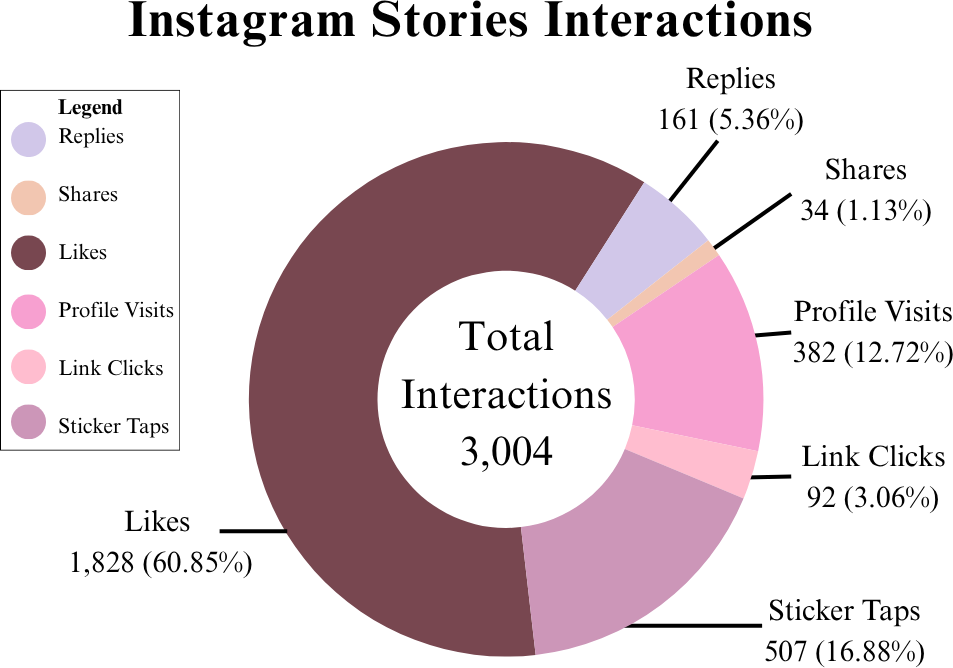

Supplement: Multimedia Appendix 12 [file formative-v10-e73445-s012.png]
