# Supplementary material for: Methodological Framework for the Design and Implementation of a US Latine-Hispanic Digital Brain Health Program: User-Centered Design Approach
Source: JMIR Form Res. 2026 May 14;10:e73445. doi: 10.2196/73445 (PMC13175527; doi:10.2196/73445)
Supplement: Multimedia Appendix 8 [file formative-v10-e73445-s008.docx]

Social Media Comments

Note. 151 comments were assessed comprising 122 total comments from Instagram (IG), 27 commnets from Facebook (FB), and 2 verbal comments from caregivers who have assessed the website. On IG, 16 comments were excluded from analysis due to being responses from team members and 2 excluded due to unavailability for a total of 18 excluded comments. 3 comments from FB were excluded from analysis due to being responses from team members.

| Comment | Hand Coder 1 | Hand Coder 2 | Combined Hand | Chat GPT | Content Description | Content Type | Platform |
| --- | --- | --- | --- | --- | --- | --- | --- |
| *Felicidades 👏👏 y muy bellas todas 😍😍🥰* [Congratulations and they are all very beautiful] | Positive | Positive | Positive | Positive | Team Intro | Carousel | IG |
| 🙌🙌🙌 | Positive | Positive | Positive | Positive | Volunteer Intro | Carousel | IG |
| ❤️ | Positive | Positive | Positive | Neutral | Dia de Los Muertos Altar | Photo/ Image | IG |
| 😍 | Positive | Positive | Positive | Positive | Dia de Los Muertos Altar | Photo/ Image | IG |
| miss my grandma 😢❤️ | Positive | Neutral | Positive | Positive | Dia de Los Muertos Altar | Photo/ Image | IG |
| ❤️😢❤️❤️ | Positive | Positive | Positive | Positive | Quote of the Month | Carousel | IG |
| 👏 | Positive | Positive | Positive | Positive | Singing and the Brain | Reel | IG |
| *Con la mejor canción! 🇵🇷🇵🇷🇵🇷* [With the best song! 🇵🇷🇵🇷🇵🇷] | Positive | Positive | Positive | Positive | Singing and the Brain | Reel | IG |
| ❤️ 🇵🇷 ❤️ 🇵🇷 ❤️ 🙌🏾 🙌🏾 🙌🏾 | Positive | Positive | Positive | Positive | Singing and the Brain | Reel | IG |
| ❤️❤️❤️ | Positive | Positive | Positive | Positive | Singing and the Brain | Reel | IG |
| ❤️❤️❤️❤️❤️ | Positive | Positive | Positive | Positive | Singing and the Brain | Reel | IG |
| Thank you!! You’re presence was so appreciated at the Alzheimer’s Association and Diane E. Watson Center for Compassionate Intervention 👏😍 | Positive | Positive | Positive | Positive | Visual Agnosia | Carousel | IG |
| *Si, somos un grupo de mujeres Poderosas, llenas de entusiasmo y motivación para seguir enfrentando a la vida antes y después de los retos de esta enfermedad terrible (Alzheimer) con amor, paciencia, dedicación, esfuerzo y fortaleza. Únete a nosotros cada semana los* *lunes de 1:30 - 3:00 pm* [Yes, we are a group of powerful women, full of enthusiasm and motivation to continue facing life before and after the challenges of this terrible disease (Alzheimer's) with love, patience, dedication, effort, and strength. Join us every week on Mondays from 1:30 to 3:00 p.m.] | Neutral | Neutral | Neutral | Positive | Caregiver Appreciation | Reel | IG |
| *Que lindo video*💞 [What a lovely video] | Positive | Positive | Positive | Positive | Caregiver Appreciation | Reel | IG |
| Beautiful, smart and brave ❤️ | Positive | Positive | Positive | Positive | Caregiver Intro | Reel | IG |
| *¡¡Que linda nombre de usuario censurado! Te admiro tanto por tu gran labor y entrega, siempre con esa linda sonrisa, que Dios siempre te cuide y bendiga con salud* [How lovely, *redacted username*! I admire you so much for your hard work and dedication, always with that beautiful smile. May God always watch over you and bless you with good health.] | Positive | Positive | Positive | Positive | Caregiver Intro | Reel | IG |
| 🙌🙌❤️❤️❤️ | Positive | Positive | Positive | Positive | Caregiver Self-Care | Carousel | IG |
| *Así es, agradecida de compartir en este grupo de apoyo, que me a brindado un increíble soporte… y si me siento bendecida y agradecida* [That's right, I am grateful to be part of this support group, which has given me incredible support... and I feel blessed and grateful.] | Positive | Positive | Positive | Positive | Thanksgiving Gratitude | Carousel | IG |
| *Gracias por crear este espacio en el para los familiares y cuidadores con un paciente de Alzheimer. Excelente medio para aprender y sentirse apoyado.* [Thank you for creating this space for family members and caregivers of Alzheimer's patients. It's an excellent way to learn and feel supported.] | Positive | Positive | Positive | Positive | Caregiver Resources | Carousel | IG |
| *Priorizar el autocuidado es un acto de amor tanto para el cuidador como para quienes cuidamos. De esta manera podremos transitar el camino con energía y salud❤️. Gracias por compartirlo* [Prioritizing self-care is an act of love for both the caregiver and those we care for. This way, we can walk the path with energy and health. Thank you for sharing.] | Positive | Positive | Positive | Positive | Quote of the Month | Carousel | IG |
| *Siiii👏👏👏 gracias* [Yes, thank you] | Positive | Positive | Positive | Positive | Community Thank You | Reel | IG |
| 🙌🙌 | Positive | Positive | Positive | Positive | Community Thank You | Reel | IG |
| ❤️❤️❤️❤️❤️ | Positive | Positive | Positive | Positive | Alzheimer's Information | Reel | IG |
| *Hermosa mi reina* 🥰 [Beautiful, my queen] | Positive | Irrelevant | Positive | Positive | Medi-cal Information | Reel | IG |
| you should follow *redacted username* you'd love her content ❤️ | Neuutra | Irrelevant | Irrelevant | Positive | Early Detection | Reel | IG |
| 🙌❤️ | Positive | Positive | Positive | Positive | Early Detection | Reel | IG |
| ❤️ *excelente, gracias por compartir* [Excellent, thank you for sharing.] | Positive | Positive | Positive | Positive | Movement and the Brain | Reel | IG |
| D'M_me_pls➢ [@*redacted*](https://www.instagram.com/blkbusinessmind/)*username* | Irrelevant | Irrelevant | Irrelevant | Irrelevant | Black History Month | Post | IG |
| D͙M͙_me ♂️@ [*redacted*](https://www.instagram.com/blkbusinessmind/)*username* | Irrelevant | Irrelevant | Irrelevant | Irrelevant | Black History Month | Post | IG |
| This is beautiful Mirella!!! Needs to go viral! You are such a shining star and example for so many! Keep knocking down doors and showing up exactly as you are! 🧠 💪🏽 💅 🖤🤎🇵🇷 | Positive | Positive | Positive | Positive | Black History Month | Reel | IG |
| [@](https://www.instagram.com/naimeh9/)[*redacted*](https://www.instagram.com/blkbusinessmind/)*username* Thank you for your support! Yes and yes 🙌🏽 Authenticity is a act of resistance. [ELHA Response] | Excluded | Excluded | Excluded | Excluded | Black History Month | Reel | IG |
| Thank you for leadership and all that you do for the community Doctora Diaz-Santos!! 👏 | Positive | Positive | Positive | Positive | Black History Month | Reel | IG |
| [@](https://www.instagram.com/salvad0or/)[*redacted*](https://www.instagram.com/blkbusinessmind/)*username* Gracias for your kind words! [ELHA Response] | Excluded | Excluded | Excluded | Excluded | Black History Month | Reel | IG |
| ❤🙌 Did you know there are over 800+ALW Approved Facilities in Southern California that accept the California Medi-Cal Assisted Living Waiver? Memory Care for Alzheimer’s or Dementia diagnosed residents, TBI Care, Assistance for those with disabilities, Mental Health Assistance and so much more in a beautiful Assisted Living Facility for literally only $1,398.07 per month. 💕 WE WILL COME TO YOU and help you get ALW Approved. We will even provide you with the tools to increase your social security to $1,575.07 per month to cover it, after move in. *Redacted Contact Information.* Get those you love the care they need.🏠❤️ | Irrelevant | Irrelevant | Irrelevant | Irrelevant | Black Researcher Highlight | Carousel | IG |
| 👏👏❤️ | Positive | Positive | Positive | Positive | New Year Goals Check-in | Reel | IG |
| 👏👏❤️ | Positive | Positive | Positive | Positive | New Year Goals Check-in | Reel | IG |
| I can't focus on that 🔥playlist + food at the same time! 😍 | Positive | Positive | Positive | Positive | Cultural Foods | Reel | IG |
| [*@nombredeusuarioredactado*](https://www.instagram.com/abc7anabel/) *ya tu sabe* 😂🔥❤ [@*redactedusername* you know] [ELHA Response] | Excluded | Excluded | Excluded | Excluded | Cultural Foods | Reel | IG |
| ❤️‍🔥 | Positive | Positive | Positive | Positive | Memory, Love, Heritage | Reel | IG |
| 👏👏 | Positive | Positive | Positive | Positive | Memory, Love, Heritage | Reel | IG |
| 👏❤️✨ | Positive | Positive | Positive | Positive | Memory, Love, Heritage | Reel | IG |
| 🧠 | Neutral | Neutral | Neutral | Neutral | Memory, Love, Heritage | Reel | IG |
| Comment Unavailable | Excluded | Excluded | Excluded | Excluded | Memory, Love, Heritage | Reel | IG |
| yay Jasmine!!! 🥳🥳 | Positive | Positive | Positive | Positive | February recap | Reel | IG |
| *Me encanto la conversa. Gracias por el apoyo a la comunidad hispana.❤*️[I loved the conversation. Thank you for supporting the Hispanic community. ️] | Positive | Positive | Positive | Positive | Podcast Introduction | Reel | IG |
| Love it! Unity takes work🙌 | Positive | Positive | Positive | Positive | Academia Poem | Reel | IG |
| 🔥🔥🔥❤❤❤ | Positive | Positive | Positive | Positive | Academia Poem | Reel | IG |
| ❤❤❤ | Positive | Positive | Positive | Positive | Academia Poem | Reel | IG |
| ❤️👏👏🥰 | Positive | Positive | Positive | Positive | Academia Poem | Reel | IG |
| Thank you for coming 💙 | Positive | Positive | Positive | Positive | ELHA in Community | Reel | IG |
| @*redactedusername* Thank you for having us #brainhealth #latinidad #endalz💜 [ELHA Response] | Excluded | Excluded | Excluded | Excluded | ELHA in Community | Reel | IG |
| A must read. Caregiver Chronicles Loving Frank, Loving Me By Enma Espinoza A Latina Published Author Amazon paperback to buy https:// www.amazon.com/dp/ 1735436453 My story is about unconditional Love and Self-care | Neutral | Irrelevant | Neutral | Irrelevant | Book Club Intro | Reel | IG |
| Omg!! I’m so grateful. Thank you so much! I hope it’s nourishing for you and the rest of the book club!! 🤍✨ | Positive | Positive | Positive | Positive | Book Club Intro | Reel | IG |
| Thank you!!!💜 Our team is super excited for this read. It perfectly aligns with our mission of transforming mind, body and spirit with community for a stronger brain 🧠 #End #Alzheimers [@](https://www.instagram.com/raquelreichard/)*redactedusername* [@](https://www.instagram.com/r29somos/)*redactedusername* [ELHA Response] | Excluded | Excluded | Excluded | Excluded | Book Club Intro | Reel | IG |
| ❤ *estoy muy orgullosa de ti!! Sigue adelante y me gustaría escuchar más de ti sobre la salud!!* 👏🏼👏🏼👏🏼 [ I am very proud of you!! Keep going, and I would like to hear more from you about health!!] | Positive | Positive | Positive | Positive | Women’s Health | Reel | IG |
| [@](https://www.instagram.com/msalcalac/)*redactedusername* thank you Claudia! Definitely stay tuned on this page for more. We do our best to break down the science so that it’s easier to bring these conversations to our communities and families! Sending my best 🥰 [ELHA Response] | Excluded | Excluded | Excluded | Excluded | Women’s Health | Reel | IG |
| *Si es cierto mi vida. Tu abuelita tiene deabetis y nosotros nos cuidamos y asta ahorita yo no he salido con nada ni colesterol gracias a Dios. 🥰🙏🏻*[Yes, it's true, my dear. Your grandmother has diabetes, and we take care of ourselves, and so far, thank God, I haven't developed any problems, not even high cholesterol.] | Positive | Positive | Positive | Positive | Women’s Health | Reel | IG |
| 👏👏👏 | Positive | Positive | Positive | Positive | Academic Representation | Reel | IG |
| 🧠🧠🧚🏽🧚🏽‍♂️ | Neutral | Neutral | Neutral | Neutral | Belonging and the brain | Reel | IG |
| 👏🏽👏🏽👏🏽 | Positive | Positive | Positive | Positive | Belonging and the brain | Reel | IG |
| [@](https://www.instagram.com/thecaregivercomadre/)*redactedusername* ❤ [ELHA Response] | Excluded | Excluded | Excluded | Excluded | Belonging and the brain | Reel | IG |
| Thank you so much for highlighting our work! ❤️ | Positive | Positive | Positive | Positive | Social connection and the brain | Reel | IG |
| It is amazing to hear that our brain can have great changes and improve our health by investing so little in money but so much in social connection. | Neutral | Neutral | Neutral | Positive | Social connection and the brain | Reel | IG |
| [*@nombredeusuarioredactado*](https://www.instagram.com/duvraskamendoza/) *Asi mismo es!* [@*redactedusername* That's right!] [ELHA Response] | Excluded | Excluded | Excluded | Excluded | Social connection and the brain | Reel | IG |
| AMen! So common with Latinos. Keep educating la gente [people] | Positive | Positive | Positive | Positive | High Blood Pressure | Reel | IG |
| 👏👏 | Positive | Positive | Positive | Positive | High Blood Pressure | Reel | IG |
| Ahhh!! Thank you so, so much! I’m so glad you loved it. 💕💕 | Positive | Positive | Positive | Positive | Self Care for Latinas Review | Reel | IG |
| thanks for sharing this info ‼️ | Positive | Positive | Positive | Positive | Farmworker experience and the brain | Reel | IG |
| Thanks for highlighting us and these other incredible organizations! 🌎💙💚 | Positive | Positive | Positive | Positive | Volunteer opportunities to help the planet | Post | IG |
| ❤️👏👏👏🙌 | Positive | Positive | Positive | Positive | Upcoming Events | Post | IG |
| Thank you for joining us to [#TakeAMentalHealthMoment](https://www.instagram.com/explore/tags/takeamentalhealthmoment/) 💙 | Positive | Positive | Positive | Positive | Mental Health Awareness Month | Reel | IG |
| @*redactedusername* Absolutely 💯 [ELHA Response] | Excluded | Excluded | Excluded | Excluded | Mental Health Awareness Month | Reel | IG |
| ❤️ | Positive | Positive | Positive | Positive | Mental Health Awareness Month | Reel | IG |
| [@](https://www.instagram.com/yarel_ramos/) *redactedusername* [@mirella.diaz](https://www.instagram.com/mirella.diaz/) two if my faves! 🙌🙌 | Positive | Positive | Positive | Positive | Panel Promotion | Post | IG |
| Go!!!!! 🔥🔥🔥 | Positive | Positive | Positive | Positive | Panel Promotion | Post | IG |
| Comment Unavailable | Excluded | Excluded | Excluded | Excluded | Amygdala: The Alarm System | Post | IG |
| [@](https://www.instagram.com/drblancaorellana/)*redactedusername* Proud of you!!🎊 | Positive | Positive | Positive | Positive |  |  | IG |
| Muchas gracias! [Thank you very much!] More to come 🙌❤️ [ELHA Response] | Excluded | Excluded | Excluded | Excluded | Interview w/ a clinical psychologist | Reel | IG |
| Excellent conversation and advocacy for the Latino/a/e community ❤️ Buena charla. Gracias por todo su abogacía para la comunidad 👏🏽👏🏽[Good talk. Thank you for all your advocacy for the community] | Positive | Positive | Positive | Positive | Interview w/ a clinical psychologist | Reel | IG |
| It always makes me so excited to see multilingual resources in dementia! Great work friend! 💜 | Positive | Positive | Positive | Positive | Interview w/ a social worker | Reel | IG |
| [@](https://www.instagram.com/amplifyingalz/)*redactedusername* 🙌 More multilingual resources to come! [ELHA Response] | Excluded | Excluded | Excluded | Excluded | Interview w/ a social worker | Reel | IG |
| ❤️ | Positive | Positive | Positive | Positive | Interview w/ a social worker | Reel | IG |
| Thank you for the invite❤️ | Positive | Positive | Positive | Positive | Interview w/ a social worker | Reel | IG |
| [@](https://www.instagram.com/sandyalcantar2021/)*redactedusername* Such an insightful conversation! [ELHA Response] | Excluded | Excluded | Excluded | Excluded | Interview w/ a social worker | Reel | IG |
| *Muy interesante como abordar el trauma con las personas con alguna demencia* [*@mirella.diaz*](https://www.instagram.com/mirella.diaz/) *deberia de invitar la Lic. Sandy al grupo 🙌🙌=* [Very interesting how to deal with trauma in people with dementia. @mirella.diaz should invite Sandy to the group 🙌🙌=] | Neutral | Neutral | Neutral | Neutral | Interview w/ a social worker | Reel | IG |
| [*@nombredeusuarioredactado*](https://www.instagram.com/consuelocambambia/) *Por supuesto! Gracias a* [*@*](https://www.instagram.com/sandyalcantar2021/)[*nombredeusuarioredactado*](https://www.instagram.com/consuelocambambia/) *por estar con nosotras 🧠🦋❤*️ [@*redactedusername* Of course! Thank you @*redactedusername* for being with us️] [ELHA Response] | Excluded | Excluded | Excluded | Excluded | Interview w/ a social worker | Reel | IG |
| Thank you for sharing this information. 🤍🤍🤍 | Positive | Positive | Positive | Positive | ADCRC Interview | Reel | IG |
| 🙌 | Positive | Positive | Positive | Positive | ADCRC Interview | Reel | IG |
| *Que hermosa my vida estoy muy orgullosa de ti y tu hermana que Dios me las bendiga 🙏🏻🙌❤️😍🥰😘🙏*🏻[How beautiful, my dear. I am very proud of you and your sister. May God bless you both.] | Positive | Irrelevant | Positive | Positive | ADRD Awareness Month | Reel | IG |
| I never knew the difference! | Neutral | Neutral | Neutral | Neutral | Early vs. Late AD | Reel | IG |
| 🙌🏽🤞🏽 | Positive | Positive | Positive | Positive | LMSA Presentation | Post | IG |
| Thank you for having us! 🙌 [ELHA Response] | Excluded | Excluded | Excluded | Excluded | LMSA Presentation | Post | IG |
| ❤️❤️❤️❤️❤️ | Positive | Positive | Positive | Positive | HNS Award Appreciation | Post | IG |
| I could use one in South Carolina & New York 👏👏🙏🏼❤️👼🏻 | Positive | Positive | Positive | Positive | Caregiver Celebration | Reel | IG |
| 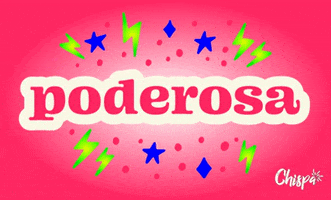[ELHA Response] | Excluded | Excluded | Excluded | Excluded | Caregiver Celebration | Reel | IG |
| My ❤️ is full! I’m not crying 🥺 you are 😂 #grateful for the trust you all have given me 🦋🪬🧠 [ELHA Response] | Excluded | Excluded | Excluded | Excluded | Caregiver Celebration | Reel | IG |
| *Gracias a usted por estar conmigo hoy ❤*️[Thank you for being with me today.] [ELHA Response] | Excluded | Excluded | Excluded | Excluded | Háblame Claro | Reel | IG |
| *Gracias por tan valiosa información Dra Mirella 🙏💕* [Thank you for such valuable information, Dr. Mirella] | Positive | Positive | Positive | Positive | Háblame Claro | Reel | IG |
| Let's dance 💃 🤪 🥳🥳 | Positive | Positive | Positive | Positive | Dancing and the Brain | Reel | IG |
| 🔥🔥🔥 | Positive | Positive | Positive | Positive | Dancing and the Brain | Reel | IG |
| *Hecho* ✔️[Done ] | Neutral | Neutral | Neutral | Neutral | Monthly Wordsearch | Post | IG |
| *Juntas son dinamita, me encantan 🙌❤️👏👏En horabuena🙌🙌*[Together they are dynamite, I love them Congratulations] | Positive | Positive | Positive | Positive | Háblame Claro | Reel | IG |
| [*@mirella.diaz*](https://www.instagram.com/mirella.diaz/) *siempre agradecida con usted por compartir tan valiosa información. 🙏*[@mirella.diaz, I am always grateful to you for sharing such valuable information.] | Positive | Positive | Positive | Positive | Háblame Claro | Reel | IG |
| ❤️👏🙌 | Positive | Positive | Positive | Positive | Elha in Community | Post | IG |
| Thank you all for the very important work you do for our communities!! ❤👏 | Positive | Positive | Positive | Positive | Elha in Community | Post | IG |
| 👏🏾 congratulations, [@elhaucla](https://www.instagram.com/elhaucla/) ! 🎉 | Positive | Positive | Positive | Positive | 1-year celebration | Reel | IG |
| *Preciosa, te escribí al interno* [Preciosa, I wrote to you internally.] | Positive | Irrelevant | Irrelevant | Irrelevant | Hablando Claro | Reel | IG |
| ❤️❤️❤️ | Positive | Positive | Positive | Positive | Volunteer Re-Introduction | Post | IG |
| 🙌🙌🙌❤️❤️ | Positive | Positive | Positive | Positive | Caregiver Group Reintroduction | Reel | IG |
| 🎄🪅🪅🪅 | Positive | Positive | Positive | Positive | Háblame Claro | Reel | IG |
| Incredibly proud of you Dr. | Positive | Positive | Positive | Positive | Háblame Claro | Reel | IG |
| You are making a difference from the ground up!! 👏🏽👏🏽 thank you for all you do!! 💕💕 | Positive | Positive | Positive | Positive | Giving Tuesday | Reel | IG |
| 👏👏👏 | Positive | Positive | Positive | Positive | Giving Tuesday | Reel | IG |
| *Te amo* [I love you] | Positive | Positive | Positive | Positive | Giving Tuesday | Reel | IG |
| Mirella- you are the best. Love following you and to see you do the work from the “ground up”. So happy to have met you and looking forward to other opportunities to work together ❤️ *Dios me la Bendiga 🙌* [God bless you] | Positive | Positive | Positive | Positive | Giving Tuesday | Reel | IG |
| 👏👏👏💯🧿 | Positive | Positive | Positive | Positive | Youtube Introduction | Reel | IG |
| Congratulations 👏👏 | Positive | Positive | Positive | Positive | Research Study Announcement | Reel | IG |
| 👏👏👏❤️ | Positive | Positive | Positive | Positive | Research Study Announcement | Reel | IG |
| 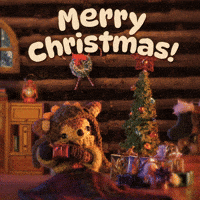 | Positive | Positive | Positive | Positive | Christmas Post | Post | IG |
| merry xmas 🎄🎅💖 | Positive | Positive | Positive | Positive | Háblame Claro | Reel | IG |
| *Gracias ❤️ feliz navidad y muchas bendiciones amén. 🙏🏻🥰🎄*[Thank you Merry Christmas and manyy blessings, amen.] | Positive | Positive | Positive | Positive | Háblame Claro | Reel | IG |
| ❤❤ | Positive | Positive | Positive | Positive | ELHA in Community | Post | IG |
| *A cantar* [To sing]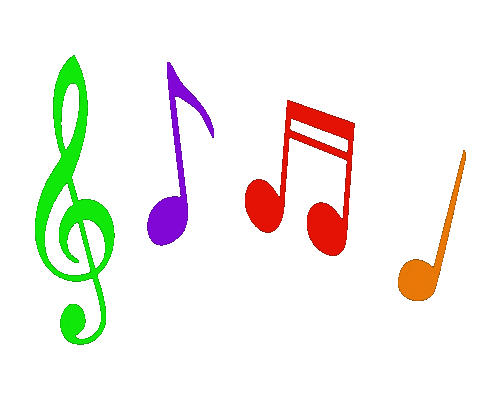 | Neutral | Neutral | Neutral | Positive | Singing and the brain | Post | FB |
| *Preciosa, Dios te Bendiga.* [Beautiful, God bless you.] | Positive | Positive | Positive | Positive | Dementia vs. Alzheimers | Post | FB |
| *Mí nieta hermosa. Dios te continúe bendiciendo y brindando conocimiento para que sigas ayudando los enfermeros.* [My beautiful granddaughter. May God continue to bless you and grant you knowledge so that you can continue to help nurses.] | Positive | Irrelevant | Positive | Positive | Dementia vs. Alzheimer | Post | FB |
| Link to a Happy New Year image | Positive | Positive | Positive | Positive | Happy New Year | Post | FB |
| *Dra. Mucha salud , y bendiciones para usted.* [Dr. Mucha, best wishes for your health and blessings to you.] | Positive | Positive | Positive | Positive | Black History Month | Video/ Reel | FB |
| Wow Doctora it makes me so angry to hear that there have been people that questioned your qualifications and how you achieved them 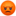 all those things you mention that supposedly make you “ different “ are in my opinion your Superpowers 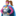 Remember the best revenge is success! 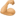Abrazos 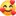 [Hugs 🥰] | Positive | Positive | Positive | Positive | Being a Woman in Academia | Video/ Reel | FB |
| *Gracias redactedusername]!* 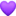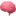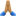 [Thank you *redactedusername]* [ELHA Response] | Excluded | Excluded | Excluded | Excluded | Being a Woman in Academia | Video/ Reel | FB |
| *Te amo mi Princesa hermosa. Té veo triste. Estoy aquí para ti. Me puedes llamar cuando quieras.* [I love you, my beautiful princess. You look sad. I'm here for you. You can call me anytime.] | Positive | Irrelevant | Positive | Positive | Deceived Poem | Video/ Reel | FB |
| My favorite neuropsychologist right here!! | Positive | Positive | Positive | Positive | Neuroscience of social connections | Video/ Reel | FB |
| *Que bella estás mi Princesa* [How beautiful you are, my Princess] | Positive | Irrelevant | Irrelevant | Positive | Cherished Memories | Video/ Reel | FB |
| *No hay online?* [Not online?] | Neutral | Neutral | Neutral | Neutral | ELHA in Community | Post | FB |
| *También hacemos IG Lives, FB Lives y grupos semanales de apoyo a cuidadores de alzheimers en español todos los lunes.* *Información sobre* *nuestros grupos de apoyo a cuidadores en Zoom se puede encontrar en nuestra página de FB* [We also host IG Lives, FB Lives, and weekly support groups for Alzheimer's caregivers in Spanish every Monday. Information about our caregiver support groups on Zoom can be found on our FB page] [ELHA Response] | Excluded | Excluded | Excluded | Excluded | ELHA in Community | Post | FB |
| *muchas gracias ! Me parece muy interesante su trabajo* [Thank you very much! I find your work very interesting.] | Positive | Positive | Positive | Positive | ELHA in Community | Post | FB |
| *¡Gracias por apoyarnos!* [Thank you for your support! ] [ELHA Response] | Excluded | Excluded | Excluded | Excluded | ELHA in Community | Post | FB |
| *Ha sido de gran placer haberlas encontrado para alimentar nuestro cerebro y trabajar en el Alzheimer también Gracias* [It has been a great pleasure to have found you to feed our brains and work on Alzheimer's as well. Thank you.] | Positive | Positive | Positive | Positive | ELHA in Community | Post | FB |
| Congrats! 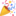 | Positive | Positive | Positive | Positive | 1 Year Anniversary | Post | FB |
| *How wonderful to have these bright young ladies on the team! Muchísimas gracias por su esfuerzo* 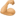 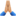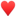[Thank you very much for your efforts.] | Positive | Positive | Positive | Positive | Volunteer Highlight | Post | FB |
| *Bravo redactedusername me gusto mucho tu plática gracias* 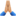 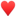[Bravo *redactedusername*, I really enjoyed your talk, thank you.] | Positive | Positive | Positive | Positive | Hablando Claro | Post | FB |
| *Hola me gustaría colaborar con ustedes* [Hello, I would like to collaborate with you.] | Positive | Neutral | Positive | Neutral | ELHA in Community | Post | FB |
| Link to Bunny rabbit clapping | Positive | Positive | Positive | Positive | Healthy Recipe | Reel | FB |
| *Gracias Siempre it más buen todos los días practico este juego me gusta tener ocupada mi mente tambien me gusta hacer cosas difíciles para tener ocupada mi mente.* [Thank you. Every day I practice this game. I like to keep my mind busy. I also like to do difficult things to keep my mind busy.] | Positive | Positive | Positive | Positive | Monthly Wordsearch | Post | FB |
| *Gracias!!!* [Thank you!!!} | Positive | Positive | Positive | Positive | Microaffirmations | Post | FB |
| *Gracias!!!* [Thanks] | Positive | Positive | Positive | Positive | Familismo and the brain | Post | FB |
| *Me encanta bailar, bailo sola!!*! [I love dancing, I dance alone!!!!] | Positive | Positive | Positive | Positive | Dancing and the Brain | Reel | FB |
| *💃 💃 a bailar se ha dicho tons. Gracias Gracias.* [a dance has been said tons. Thank you.] | Positive | Positive | Positive | Positive | Dancing and the Brain | Reel | FB |
| *Buen día mí Princesa hermosa. Dios te bendiga* [Good morning my beautiful Princess. God bless you] | Irrelevant | Irrelevant | Irrelevant | Positive | representation in academia | Reel | FB |
| Great explanation of nutrients! Gracias 🙏🏼 | Positive | Positive | Positive | Positive | nutrition | Post | FB |
| *I like the videos. For me, the videos are like experiences. When I see, I remember* | Positive | Positive | Positive | Positive | General Page | General Page | Verbal |
| Lo principal es que la información y el material ilustrado es entendible. Ayuda a los pacientes, los cuidadores, y a toda la comunidad que habla Español, a no solo entender el Alzheimer, si no también en como cuidarse, usando un lenguaje sencillo pero profundo. | Positive | Positive | Positive | Positive | General Page | General Page | Verbal |
