# Supplementary material for: Methodological Framework for the Design and Implementation of a US Latine-Hispanic Digital Brain Health Program: User-Centered Design Approach
Source: JMIR Form Res. 2026 May 14;10:e73445. doi: 10.2196/73445 (PMC13175527; doi:10.2196/73445)
Supplement: Multimedia Appendix 9 [file formative-v10-e73445-s009.docx]

| Code | Coding Description | Examples |
| --- | --- | --- |
| Irrelevant | Comments that are unrelated to the original post, the general content of the page, or the broader context. This includes:   - Responses to other comments, even if the original discussion aligns with the topic. - Spam messages, advertisements, or promotional content. | - "Check out my profile!" - "First comment!" - "This reminds me of something completely unrelated to the topic." |
| Negative | Comments or emojis expressing negative sentiment, dissatisfaction, criticism, or dislike towards the post, page, or creator.   - Direct criticism: "This post is terrible." - Dislike expressed through emojis: 👎, 😡. - Negative language or tone, including sarcasm. | - "This is misleading and wrong." - "I hate this team!" - "Why do you even post such irrelevant content?" |
| Neutral | Comments or questions seeking clarification, providing additional information without an emotional tone, or comments that cannot be categorized as clearly positive or negative.   - Information-seeking questions: "Can you provide more details about this?" - Statements of fact or observation without affective cues: "This was posted yesterday." - Ambiguous language that cannot be assigned to other categories. | - "What time does the event start?" - "Is this based on verified information?" - "Interesting post." |
| Positive | Comments or emojis that express approval, appreciation, excitement, or support for the post, page, or creator.   - Direct expressions of support: "Great post!" - Positive emojis: ❤️, 😊, 👏. - Excited or enthusiastic language: "This is amazing!" | - "Fantastic work as always!" - "Love this team! Keep it up!" - "❤️ this post!" |

Directions for ChatGPT Sentiment Analysis

- Code each comment separately
- Code for positive and negative sentiment
- Provide the coding in a spreadsheet format
- Use the codebook provided (need to discuss w/ MDS)

Citations that Inform

- [Engagement Patterns with Female and Male Scientists on Facebook](file:///Users/aishamohammed/Downloads/final.pdf)
  - <https://www.researchgate.net/figure/Codebook-for-comments-to-posts_tbl1_360900495>
- <https://www.tandfonline.com/doi/full/10.1080/19312458.2020.1869198#d1e2614>
- <https://www.researchgate.net/publication/381881700_A_review_of_sentiment_analysis_tasks_applications_and_deep_learning_techniques>
- [Public Health Discussions on Social Media: Evaluating Automated Sentiment Analysis Methods](https://www.sciencedirect.com/org/science/article/pii/S2561326X25000162#aep-e-component-id9)
  - <https://ars.els-cdn.com/content/image/1-s2.0-S2561326X25000162-formative_v9i1e57395_app1.pdf>
